# Liquid–liquid phase separation-related patterns in glioblastoma: Immune landscape, prognostic features, and therapeutic resistance

**DOI:** 10.1016/j.gendis.2025.101562

**Published:** 2025-02-18

**Authors:** Shengqi Hu, Jiajia Gao, Jing Wang, Fusheng Liu, Chun Zeng

**Affiliations:** a Department of Interventional Neuroradiology, Beijing Neurosurgical Institute, Capital Medical University, Beijing 100070, China; b Department of Neurosurgery, Beijing TianTan Hospital, Capital Medical University, Beijing 100070, China; c Brain Tumor Research Center, Beijing Neurosurgical Institute, Capital Medical University, Beijing 100070, China; d China National Clinical Research Center for Neurological Diseases, Beijing 100070, China; e Department of Neurosurgery, Peking University International Hospital, Beijing 100070, China

Glioblastoma multiforme (GBM) remains the most aggressive and challenging central nervous system tumor due to the heterogeneity of the tumor microenvironment, prompting suboptimal effects to immune checkpoint blockade treatments.^1^^,^^2^ Notably, biomolecular condensates formed via liquid–liquid phase separation (LLPS) have been implicated in cancer progression by altering the tumor microenvironment and enhancing drug resistance.^3^, ^4^, ^5^ Consequently, it is urgent and imperative to identify valuable differentially expressed LLPS-related genes (DELRGs) and establish a prognostic model for GBM based on LLPS to address the immunosuppressive tumor microenvironment in GBM. Therefore, our study is dedicated to elucidating the machinery of LLPS-related genes in GBM by analyzing transcriptomic and single-cell RNA sequencing data, supported by experimental validation. The workflow for the transcriptomic, clinical, and single-cell RNA sequencing data analysis of GBM patients based on LLPS-related genes is shown in Figure 1.

**Figure 1:**
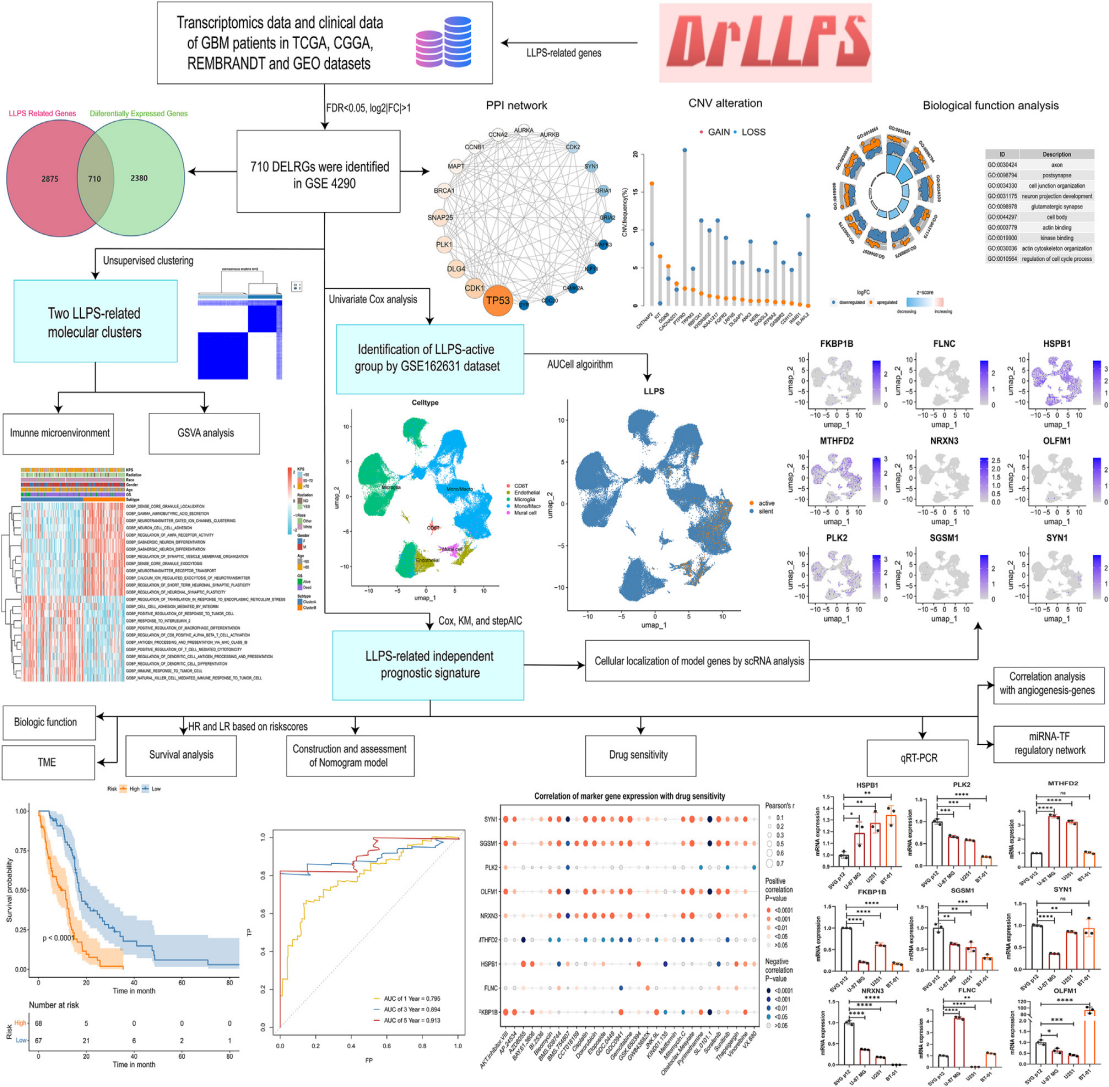
The comprehensive workflow for transcriptomic, clinical, and single-cell RNA sequencing data analysis of GBM patients based on LLPS-related genes. CGGA, Chinese Glioma Genome Atlas; CNV, copy number variations; LLPS, liquid–liquid phase separation; DELRGs, differentially expressed LLPS-related genes; FDR, false discovery rate; GBM, glioblastoma multiforme; GEO, Gene Expression Omnibus; GSVA, Gene Set Variation Analysis; HR, high risk; KM, Kaplan–Meier; LR, low risk; PPI, protein–protein interaction; TCGA, The Cancer Genome Atlas; TF, transcription factor, TME, tumor microenvironment.

We identified 710 DELRGs between GBM and normal samples from the GSE4290 dataset (Figs. S1A and B). A protein–protein interaction network revealed five potential hub genes, namely, *TP53*, *CDK1*, *DLG4*, *PLK1*, and *SNAP25* (Fig. S1C). Analysis of the molecular alterations in these DELRGs showed missense mutations to be the most common variant, with *TP53*, *RYR2*, *PCLO*, *ANK2*, and *RELN* being the top mutated genes (Fig. S1D). Copy number variation analysis showed significant alterations in the top 20 mutated DELRGs (Fig. S1E). Functional enrichment analyses linked these genes to axons, post-synapses, organization of cell junctions, and development of neuronal projections (Fig. S1F).

Unsupervised clustering of DELRG expression in 143 GBM samples identified two molecular subtypes, namely, clusters A and B (Figs. S2A–C). Gene set variation analysis revealed a relationship between these subtypes and clinical features, including Karnofsky performance score, radiation, race, sex, survival status, and age (Fig. S2D). Cluster B was mainly associated with neurotransmitter receptor transport, neuron cell adhesion, and neuronal differentiation, while cluster A was linked with immune responses, including positive regulation of responses to tumor cells, differentiation of macrophages, and T cell-mediated cytotoxicity. ESTIMATE analysis indicated that cluster A had higher immune scores. Single sample gene set enrichment analysis revealed that most innate and adaptive immune cells were more abundant in cluster A (Fig. S2F). Immune checkpoint genes, including *CD274*, *CD276 NRP1*, *TNFSF4*, *TNFRSF15*, *TNFRSF18*, *IDO1*, and *PDCD1LG2*, were strongly expressed in cluster A (Fig. S2G). Cluster B had a significantly higher stemness score (Fig. S2H).

After univariate Cox analysis, we identified 70 LLPS-related genes that were associated with the prognosis of GBM and analyzed their expression in single-cell RNA sequencing data from the GSE162631 dataset (Table S1). Twenty cell clusters and transcriptomes of five major cell types (*i.e.*, CD8^+^ T, endothelial, microglial, mono/macro, and mural) were analyzed based on the expression of gene markers (Figs. S3A and B). Assessment of LLPS activity using the AUCell package in R identified 3208 LLPS-active cells (area under the curve > 0.12) (Fig. S3C). The remaining cells were classified as LLPS-silent (Fig. S3D). LLPS genes were more active in mural cells than in other cell types (Fig. S3E).

We selected nine independent prognostic genes using Cox, Kaplan–Meier, and stepAIC methods (Tables S1–3). The risk score was calculated as follows: risk score = (0.5372) ∗ FKBP1B + (0.1147) ∗ FLNC + (0.4466) ∗ HSPB1 + (−0.4986) ∗ MTHFD2 + (0.7032) ∗ NRXN3 + (0.3202) ∗ OLFM1 + (0.2057) ∗ PLK2 + (0.7692) ∗ SGSM1 + (−0.5312) ∗ SYN1 (Fig. S4A). The transcriptional expression of the nine independent prognostic genes is shown in Figure S4B. GBM patients were classified as high-risk or low-risk based on the median risk score. Notably, in the TCGA cohort, median overall survival was significantly better in the low-risk group than in the high-risk group (16.3 *vs*. 11.4 months, *p* < 0.0001; Fig. S4B). We used three independent cohorts (REMBRANDT, CGGA325, and CGGA693) to validate the model. In all validation cohorts, median overall survival was better in the low-risk group (REMBRANDT: 18.0 *vs*. 12.5 months, *p* = 0.00065, Fig. S4C; CGGA325: 15.8 *vs*. 10.4 months, *p* = 0.0013, Fig. S4D; CGGA693: 16.4 *vs*. 10.4 months, *p* = 0.027, Fig. S4E). The distribution of risk scores and survival status in the TCGA, CGGA325, and CGGA693 cohorts is shown in Figs. S4F–H.

Univariate and multivariate Cox analyses identified risk scores from the nine-gene model, along with radiation therapy, as independent prognostic factors for GBM (Figs. S5A and B). We then developed a nomogram incorporating these factors (Fig. S5C), which showed a significant difference in median survival between the low-risk and high-risk groups (17.0 *vs*. 9.3 months, *p* < 0.0001, Fig. S5D). The nomogram model had an area under the curve of 0.795, 0.894, and 0.913 for 1-year, 3-year, and 5-year survival, respectively (Fig. S5E). The decision curve analysis confirmed the clinical utility of the nomogram at these survival intervals (Fig. S5F).

We analyzed differences in immune checkpoint expression, hypoxia scores, and genomic changes between the high-risk and low-risk groups. The high-risk group had higher *CD44*, *TNFRSF18*, *TNFRSF25*, *TNFSF9*, and *TNFSF14*, *TNFRSF18*, and *TNFRSF25* levels, while the low-risk group had a higher IDO1 level (Fig. S6A). High-risk patients also had higher hypoxia scores but lower tumor aneuploidy scores, mutation counts, and tumor mutation burden (Fig. S6B). Notably, *TP53* and *MUC16* showed inverse mutation frequencies in the high-risk and low-risk groups (28%/38% for *TP53*; 11%/18% for *MUC16*) (Fig. S6C). There were significant between-group differences in copy number variation (Fig. S6D). Given the critical role of angiogenesis in GBM malignancy, we analyzed its correlation with nine prognostic genes. *CDH13* showed strong positive correlations with *NRXN3*, *OLFM1*, *SGSM1*, and *SYN1*, but a negative correlation with *HSPB1. ATP5IF1* and *MYH9* exhibited opposite correlation patterns with *FKBP1B* and *FLNC* (Fig. S6E; Table S4).

To investigate the molecular mechanisms distinguishing the high-risk and low-risk groups, we identified 139 up-regulated and 13 down-regulated genes (Fig. S7A). Gene ontology analysis indicated that the differentially expressed genes were primarily involved in biological processes such as chemical synaptic transmission, trans-synaptic signaling, signal release, synaptic vesicle transport, and regulation of exocytosis. These genes were also associated with cellular components such as collagen trimers, dendritic spines, distal axons, neuronal cell bodies, and neuron spines. They were linked with molecular functions including calcium-dependent phospholipid and protein binding, calmodulin binding, extracellular matrix structure, and syntaxin-1 binding (Figs. S7B–D). Gene set enrichment analysis showed that homologous recombination and DNA replication were more likely in low-risk patients. The pathways related to dopaminergic synapse, GABAergic synapse, morphine addiction, extracellular matrix–receptor interaction, focal adhesion, motor proteins, and tumor necrosis factor signaling are enriched in high-risk patients (Figs. S7E and F).

Assessment of the relationship between the prognostic model and drug sensitivity (Fig. S8A) identified resistance to cisplatin, doxorubicin, etoposide, bleomycin, gemcitabine, and sorafenib in high-risk patients (Figs. S8B–G).

Single-cell RNA sequencing analysis of GBM revealed the expression patterns of the nine prognostic LLPS genes, with obvious *HSPB1*, *PLK2*, and *MTHFD2* expression (Fig. S9). We also constructed a transcription factor-miRNA coregulatory network that identified 59 transcription factor genes and 192 miRNAs that interacted with these prognostic LLPS genes (Fig. S10 and Table S5).

Quantitative reverse transcription PCR assays in human glioma cell lines confirmed the predicted expression patterns of nine independent LLPS-related hub genes: namely, low *PLK2*, *FKBP1B*, *SGSM1*, *OLFM1*, *NRXN3*, and *SYN1* levels and high *FLNC HSPB1* and *MTHFD2* levels (Fig. S11). These results were consistent with RNA sequencing results from the GSE4290 (Fig. S4B), further validating the prognostic significance of these genes in GBM.

In summary, we identified the critical role of LLPS-related genes in GBM, focusing on their influence on the tumor microenvironment, immune regulation, and drug resistance. A nine-gene LLPS-based prognostic model was developed, effectively stratifying patients into high-risk and low-risk groups with significant survival differences, supported by transcriptomic, single-cell RNA sequencing, and experimental validation. The explicit relationship between nine hub genes and the immune-heterogenous tumor microenvironment needs further fundamental research and clinical trials.

## CRediT authorship contribution statement

**Shengqi Hu:** Writing – original draft, Conceptualization. **Jiajia Gao:** Writing – original draft, Conceptualization. **Jing Wang:** Writing – original draft, Conceptualization. **Fusheng Liu:** Writing – review & editing, Supervision, Conceptualization. **Chun Zeng:** Writing – review & editing, Supervision, Conceptualization.

## Conflict of interests

The authors have no conflict of interests to declare.
