# Supplementary material for: Liquid–liquid phase separation-related patterns in glioblastoma: Immune landscape, prognostic features, and therapeutic resistance
Source: Genes Dis. 2025 Feb 18;13(1):101562. doi: 10.1016/j.gendis.2025.101562 (PMC12494542; doi:10.1016/j.gendis.2025.101562)
Supplement: Multimedia component 1 [file mmc1.docx]

**MATERIALS AND METHODS**

**Data Acquisition and Preprocessing**

Bulk RNA sequencing data and clinical annotations of GBM patients were obtained from The Cancer Genome Atlas (TCGA) in the UCSC Xena project and also used as the training cohort. A total of 143 GBM samples were obtained. The REMBRANDT (n=210 samples), CGGA 325 (n=137 samples), and CGGA 693 (n=237 samples) datasets were downloaded from the Gene Expression Omnibus (GEO) and the Chinese Glioma Genome Atlas (CGGA)for further gene expression profiling and survival analyses of the corresponding patients in external validation. The GSE4290 dataset (77 tumor, 23 normal) for differential analysis and human GBM single-cell RNA-sequencing dataset GSE162631 was all obtained from the GEO. Subsequently, a total of 3,585 LLPS-related genes were downloaded from DrLLPS.

**Differentially Expressed Gene Analysis and Functional Analysis**

The R package “limma” was used to identify DELRGs between GBM cases and normal cases in the GEO4290 dataset. The cut-off value was set at false discovery rate (FDR) < 0.05 and log2 |fold change (FC)| > 1. The Metascape database was applied for the gene ontology (GO) and Kyoto Encyclopedia of Genes and Genomes (KEGG) enrichment analyses about collected DELRGs, with an adjusted P value < 0.05 considered statistically significant.

**Identification of liquid–liquid phase separation-related subtypes**

The R package 'ConsensusClusterPlus' was subsequently used to identify LLPS-related molecular clusters. Furthermore, the correlations between these clusters and the clinicopathologic characteristics and survival of GBM patients were explored. Using the ESTIMATE method and the Single-sample Gene Set Enrichment Analysis (ssGSEA) algorithm, the dissimilarity of the immune microenvironment and immune infiltrating cells among different LLPS-related molecular clusters were particularly revealed. Hypoxia scores and gene mutation data of GBM were obtained from cBioPortal.

Tumor stemness analysis was performed on 26 stemness gene sets using the web-based tool StemChecker (http://stemchecker.sysbiolab.eu/), based on the most comprehensive and updated collection of published stemness signatures. The ssGSEA was implemented to quantitatively elucidate the stemness enrichment scores of the 26 stemness gene sets in each GBM sample via the Gene Set Variation Analysis (GSVA) R package, and differential expression gene (DEG) analysis was performed in both groups.

GSVA was used to investigate the potential mechanisms between LLPS-related molecular clusters, with a significance threshold of FDR < 0.05.

**Development of the LLPS scoring system**

LLPS-related prognostic genes were identified from DELRGs using univariate Cox proportional hazards analysis based on a threshold of *p* < 0.05. Additionally, Kaplan–Meier survival analysis (*p* < 0.05) and stepwise Akaike information criterion (stepAIC) with the MASS package were conducted to determine the most contributable LLPS genes to GBM prognosis. LLPS genes with the best predictive value were then entered into the multivariate Cox proportional hazards regression model. The LLPS scoring system was developed using a linear combination of the regression coefficients from the multivariate Cox proportional hazards model, multiplied by normalized LLPS gene expression levels:

$$Risk score=\sum_{\mathcal{i}=1}^{N} (E\mathcal{x}p\mathcal{i}\times Coei)$$

GBM patients were divided into high-risk (HR) and low-risk (LR) groups based on the median cutoff value. Subsequently, using the "survminer," "survival," and "survivalROC" R packages, Kaplan-Meier and ROC curve analyses were performed to assess the prognostic performance of the novel gene signature. Finally, univariate and multivariate Cox regression analyses were carried out to estimate the prognostic independence of the LLPS-related risk score and other clinical parameters in GBM patients. To predict survival, a nomogram model was established by including significant risk factors. The precision of the nomogram was assessed using decision curve analysis (DCA).

**Association of the LLPS-related signature with biological heterogeneity**

To understand the heterogeneity between risk groups, genomic alterations, gene expression changes, the immune microenvironment, hypoxia status, tumor stemness scores, and biological functions were compared. Gene mutations and copy number variations (CNVs) were analyzed using the maftools R package and GISTIC2.0. In addition, we extracted the immune checkpoint related gene expression matrix and performed DEG analysis for additional analysis. The hypoxia scores of GBM were obtained from cBioPortal. Drug sensitivity prediction was conducted using “oncoPredict”. The angiogenesis-related gene set was obtained from the Molecular Signatures Database (MSigDB) and processed using the R packages GSVA and GSEABase to extract gene IDs. Pearson correlation coefficients were calculated and visualized as a heatmap using the pheatmap package.

The “limma” R package was used to explore DEGs between risk groups, using |log2 FC| > 0.5 and P value < 0.05 as cut-off values. To obtain the biological enrichment entries, GO enrichment analysis was performed using the clusterProfiler R package based on DEGs between risk groups (FDR < 0.05). Gene Set Enrichment Analysis (GSEA) of KEGG pathways between the LR and HR groups was conducted using the “clusterProfiler” R package The threshold for significantly enriched pathways was set at FDR < 0.05.

**Single-cell RNA-seq (scRNA-seq) data analysis**

We downloaded GBM single-cell sequencing data containing four samples of GBM patients from GSE162631. The scRNA-seq data were processed and analyzed using the R package “Seurat”. Cells with fewer than 200 or more than 5,000 genes and mitochondrial gene fragments greater than 10% were filtered. The count data were then normalized and scaled using Seurat’s NormalizeData() and ScaleData() functions. Dimension reduction and cluster identification of cells were implemented using the RunUMAP() and FindClusters() functions. Different cell clusters were then annotated according to the data in the original study. The 'FeaturePlot' function in the Seurat R package was used to show the expression of specific genes.

The AUCell algorithm is an innovative computational method used in the realm of single-cell RNA-seq data analysis. Its primary goal is to score individual cells based on predefined sets of genes, with an emphasis on gene expression rankings rather than absolute expression levels The function “AUCell_exploreThresholds” was used to calculate the threshold to consider the current gene set active.

**Cell lines and cell culture**

Human brain astrocyte SVG p12 cells were kindly provided by the Cell Bank of the Chinese Academy of Sciences. U-87 MG and U251 MG glioblastoma cell lines were obtained from the Cell Resource Center of Peking Union Medical College (PCRC). BT-01 cells were kindly provided by Prof. Fusheng Liu . Authentications for above cell lines acquired by short tandem repeat (STR) profiling. Cells were all cultured in Dulbecco's modified Eagle medium (Gibco, USA) containing 10% fetal bovine serum (Gibco, USA) and were incubated at 37 °C in 5% CO_2_.

**Real-time reverse transcription polymerase chain reaction (qRT-PCR)**

Total cellular RNA was isolated using TRIzol reagent (Biosharp, BS258A) according to the manufacturer's instructions. The purity and concentration of RNA wereevalutaed with a NanoDrop™ OneC Spectrophotometer (ThermoFisher Scientific, Madison, WI, USA). 1 µg total RNA was reverse transcribed into cDNA using All-in-One First-Strand Synthesis MasterMix (Lablead F0202, Beijing, China) according to the standard protocol. The following human primers were generated: FKBP1B, forward 5ʹ-TGCTCCAAAATGGGAAGAAGT-3ʹ, reverse 5ʹ-GCTGCACCCTCTTCAAAACC-3ʹ; FLNC, forward 5ʹ-ATGGTAGCTGCACCGTGGAGTA-3ʹ, reverse 5ʹ-TCCACCACATCCTTCACTGGCA-3ʹ; HSPB1, forward 5ʹ-CTGACGGTCAAGACCAAGGATG-3ʹ, reverse 5ʹ-GTGTATTTCCGCGTGAAGCACC-3ʹ; MTHFD2, forward 5ʹ-GGGAAGAATGTGGTTGTGGC-3ʹ, reverse 5ʹ-ATGACTGCTGCTCCTTCCTT-3ʹ; NRXN3, forward 5ʹ-CATTGCAGTCGAGCTTGTCAAGG-3ʹ, reverse 5ʹ-CCGAGTGATGACGACATTGTGC-3ʹ; PLK2, forward 5ʹ-GCTGATGTCTGGCTGTTCATCAG-3ʹ, reverse 5ʹ-CTTCCCTGTAGATCTCACAGTG-3ʹ; OLFM1, forward 5ʹ-CAACAAGTTCCAGAGCCACA-3ʹ, reverse 5ʹ-CCAGCCTACTGACCACGA-3ʹ; SGSM1, forward 5ʹ-CATCATGTGCAGCTACATCTG-3ʹ, reverse 5ʹ-CTGATGCATCAGCTCAAACAG-3ʹ; SYN1, forward 5ʹ-TACCCCGTGGTTGTGAAGAT-3ʹ, reverse 5ʹ-GTCCTGGAAGTCATGCTGGT-3ʹ; Actin-β, forward 5ʹ-TTGTTACAGGAAGTCCCTTGCC-3ʹ, reverse 5ʹ-ATGCTATCACCTCCCCTGTGTG-3ʹ. qRT-PCR was performed on triplicate samples using a QuantStudio™ 5 (Applied Biosystems, Waltham, MA, USA) with 2× RealStar Fast SYBR qPCR Mix (GenStar A304, Beijing, China). Actin-β was used as the internal standard. Results were calculated as 2−ΔΔCt.

**Statistical Analysis**

We used R software (v4.3.1) to perdorm most statistical analyses. The Wilcoxon test was used for pairwise comparisons between two groups, and the Kruskal–Wallis test was used for multiple group comparisons. The Kaplan–Meier method and log-rank test were performed for survival analysis. The qRT-PCR data were analyzed using unpaired two-tailed t-tests in GraphPad Prism 8.0 software. Experiment data are presented as the mean ± standard deviation (SD) and are derived from at least three independent replicate experiments. A p-value of < 0.05 was considered statistically significant. (* p < 0.05; ** p < 0.01; *** p < 0.001; **** p < 0.0001)

**Supplementary Figures and Tables**

**Supplementary Figure legends:**

**Supplementary Figure 1(Figure S1):** Variant landscape of LLPS related genes in GBM patients. (A) Volcano plot of the DEGs in GBM (blue: down-regulated DEGs; orange: up-regulated DEGs; grey: unchanged genes), FDR < 0.05 and |log2FC| > 1. (B) Venn diagram between GBM DEGs and LLPS related genes. (C) The PPI network of the DELRGs associated proteins. (D) An oncoplot of the molecular alteration landscape of DELRGs in GBM. (E) The top 20 mutated DELRGs exhibited significant CNV alterations. (F) GO and KEGG enrichment analyses of DELRGs in GBM. Abbreviations: LLPS, Liquid-Liquid Phase Separation; GBM, Glioblastoma multiforme; DEGs, differentially expressed genes; FDR, False Discovery Rate; PPI, protein-protein interaction; DEKRGs, differentially expressed LLPS related genes; CNV, copy number variations; GO, gene ontology; KEGG, Kyoto Encyclopedia of Genes and Genomes.

**Supplementary Figure 2(Figure S2):** Unsupervised clustering of 710 DELRGs in GBM and characteristics of two distinct LLPS related subtypes in GBM. (A) 143 samples from the TCGA GBM cohort were divided into two clusters using a consensus clustering algorithm (k=2). (B) Cumulative Distribution Function from k=2 to 10. (C) Principal Component Analysis shows significant differences between the two LLPS clusters. (D) Heatmap shows differences in clinical information and prognostic LLPS-related DEGs expression between the two LLPS clusters. (E) The violin plot shows higher immune infiltration, stromal, and ESTIMATE score and lower tumor purity in Cluster A. (F) The boxplot of 28 types of infiltrated immune cell types. (G) Box plot of expression levels of immune checkpoint-associated genes in two LLPS related subtypes. (H) Box plot displaying the differences of 26 ssGSEA stemness scores between two LLPS related subtypes. Abbreviations: LLPS, Liquid-Liquid Phase Separation; DELRGs, differentially expressed LLPS related genes; GBM, Glioblastoma multiforme; TCGA, The Cancer Genome Atlas; ssGSEA, Single-sample Gene Set Enrichment Analysis.

**Supplementary Figure 3(Figure S3):** Sing-cell RNA-sequencing analysis of 70 LLPS-related genes associated with prognosis in GBM. (A-B) Unsupervised clustering analysis and cell types of four brain glioblastoma samples from GSE162631 in Seurat. (C) Violin plot of LLPS activity of 70 LLPS-related prognostic genes in five major cell subgroups. (D) AUC histogram displays the number of cells with an AUC value > 0.12 was 3,208. (E) The distribution of LLPS-active and LLPS-silent genes in each cell subset. Abbreviations: LLPS, Liquid-Liquid Phase Separation; AUC, area under the curve.

**Supplementary Figure 4(Figure S4):** Construction of a LLPS related prognostic signature for GBM patients. (A) Kaplan-Meire survival analysis of nine prognostic DELRGs in GBM. (B) The transcriptional expression of the nine independent prognostic genes; (C-F) Overall survival in the low- and high-risk score group patients in TCGA-GBM, REMBRANDT, CGGA325 and CGGA693. (G-I) The distribution of risk scores and survival status in low- and high-risk score group in the TCGA, CGGA325, and CGGA693 cohorts. Abbreviations: LLPS, Liquid-Liquid Phase Separation; DELRGs, differentially expressed LLPS related genes; GBM, Glioblastoma multiforme; TCGA, The Cancer Genome Atlas; CGGA, Chinese Glioma Genome Atlas.

**Supplementary Figure 5(Figure S5):** Establishment and assessment of the nomogram survival model. (A) Univariate analysis for the clinicopathologic characteristics and risk score in TCGA-GBM. (B) Multivariate analysis for the clinicopathologic characteristics and risk score in TCGA-GBM. (C) A nomogram was established to predict the prognostic of GBM patients. (D) Kaplan-Meier analyses for the two GBM groups based on the nomogram score. (E) Receiver operating characteristic curve of the combined model for 1-, 3- and 5-year survival in TCGA-GBM. (F) Decision curve analysis of nomogram predicting 1-, 3-, and 5-year overall survival. Abbreviations: GBM, Glioblastoma multiforme; TCGA, The Cancer Genome Atlas.

**Supplementary Figure 6(Figure S6):** Analysis of tumor microenvironment of LLPS-related prognostic signature. (A) Violin plot analysis of immune checkpoints including CD44, IDO1, TNFRSF18, TNFRSF25, TNFSF9 and TNFSF14 in LR and HR group. (B) Violin plot shows difference analysis of tumor mutation burden (TMB), mutation count, tumor aneuploidy score and hypoxic score in HR and LR group. (C) Oncoplot of difference analysis of molecular mutation in HR and LR groups. (D) Difference of CNV analysis in LR and HR groups. (E) the heatmap of correlation analysis between the angiogenesis-related genes and nine LLPS-related independent prognostic genes. Abbreviations: LLPS, Liquid-Liquid Phase Separation; LR, low-risk; HR, high-risk.

**Supplementary Figure 7(Figure S7):** Biologic functions underlying biological characteristics of LLPS related subtypes. (A) Volcano plot displaying DEGs (FDR < 0.05 and |log2FC| > 1) between HR and LR groups. (B) Bubble plot showing biological process of DEGs in GO enrichment analysis. (C) Network diagram showing cellular component of DEGs in GO enrichment analysis. (D) Circle plot showing molecular function of DEGs in GO enrichment analysis. (E-F) GSEA enrichment analysis of LR and HR patients. Abbreviations: DEGs, differentially expressed genes; FDR, FDR, False Discovery Rate; LR, low-risk; HR, high-risk; GO, gene ontology; GSEA, Gene Set Enrichment Analysis.

**Supplementary Figure 8(Figure S8):** Drug sensitivity analysis of LLPS-related prognostic signature. (A) The landscape of drug sensitivities for LLPS-related prognostic model. (B-G) IC50 values of Cisplatin, Doxorubicin, Etoposide, Bleomycin, Gemcitabine, and Sorafenib in LR and HR group. Abbreviations: LLPS. Liquid-Liquid Phase Separation; LR, low-risk; HR, high-risk.

**Supplementary Figure 9(Figure S9):**  The expression of nine LLPS-related prognostic genes in the tumor microenvironment. (A) The distribution of prognostic LLPS genes in each cell subset. (B) Bubble plot of the expression of nine LLPS-related prognostic genes including FKBP1B, FLNC, HSPB1, MTHFD2, NRXN3, PLFM1, PLK2, SGSM1 and SYN1 in CD8^+^T, endothelial, microglia, mono/macro, mural cells. Abbreviations: LLPS, Liquid-Liquid Phase Separation.

**Supplementary Figure 10(Figure S10):** The transcription factor -miRNA coregulatory network, and a total of 59 transcription factor genes and 192 miRNAs interacted with the nine prognostic liquid-liquid phase separation genes.

**Supplementary Figure 11(Figure S11):** The mRNA expression levels of prognosis model related hub genes. (A-F) qRT-PCR assay displaying the different mRNA expression of FKBP1B, HSPB1, MTHFD2, PLK2, SGSM1, FLNC, NRXN3, OLFM1 and SYN1 in SVG p12 cells and GBM cells. Statistical differences between the groups were analyzed using unpaired two-tailed t-tests (* *p* < 0.05; ** *p* < 0.01; *** *p* < 0.001; **** *p* < 0.0001). All data are presented as the mean ± standard deviation (SD) and represented at least three independent replicate experiments.

**Supplementary Table 1 (Table S1):** Significant genes of differentially expressed liquid-liquid phase separation related genes in univariate COX regression.

**Supplementary Table 2 (Table S2):** Significant prognostic genes of liquid-liquid phase separation -related genes in the Kaplan–Meier survival analysis.

**Supplementary Table 3 (Table S3):** The multivariate results of liquid-liquid phase separation -related prognostic genes.

**Supplementary Table 4 (Table S4):** Results of correlation analysis between LLPS-related independent prognostic genes with angiogenesis-related genes.

**Supplementary Table 5 (Table S5):** The intersection of the nine prognostic genes with 59 transcription factor genes and 192 miRNAs.
