# Supplementary figures and images for: Liquid–liquid phase separation-related patterns in glioblastoma: Immune landscape, prognostic features, and therapeutic resistance
Source: Genes Dis. 2025 Feb 18;13(1):101562. doi: 10.1016/j.gendis.2025.101562 (PMC12494542; doi:10.1016/j.gendis.2025.101562)

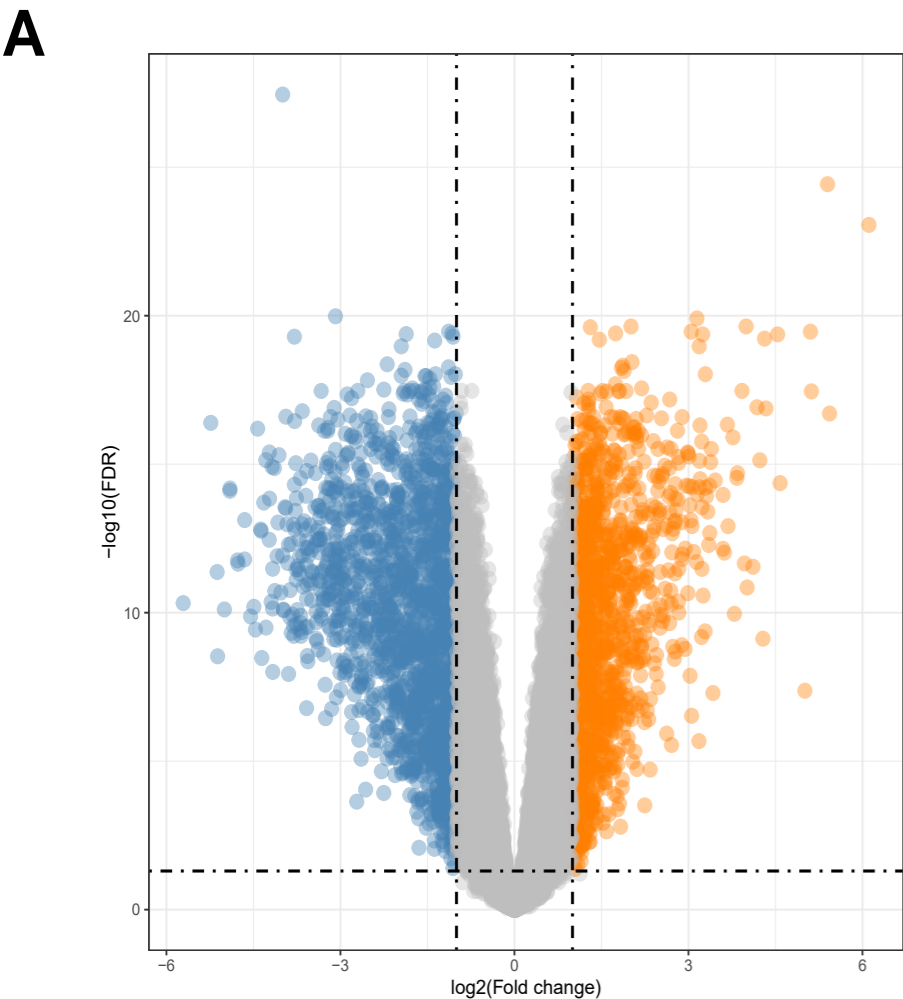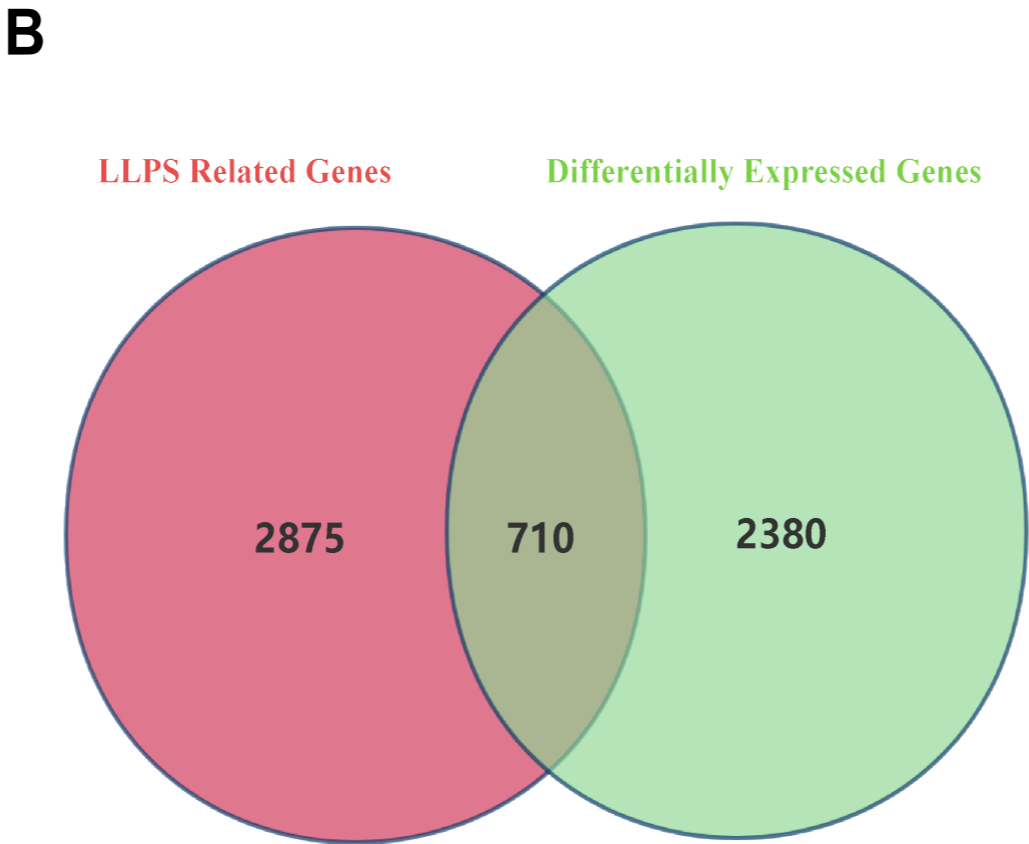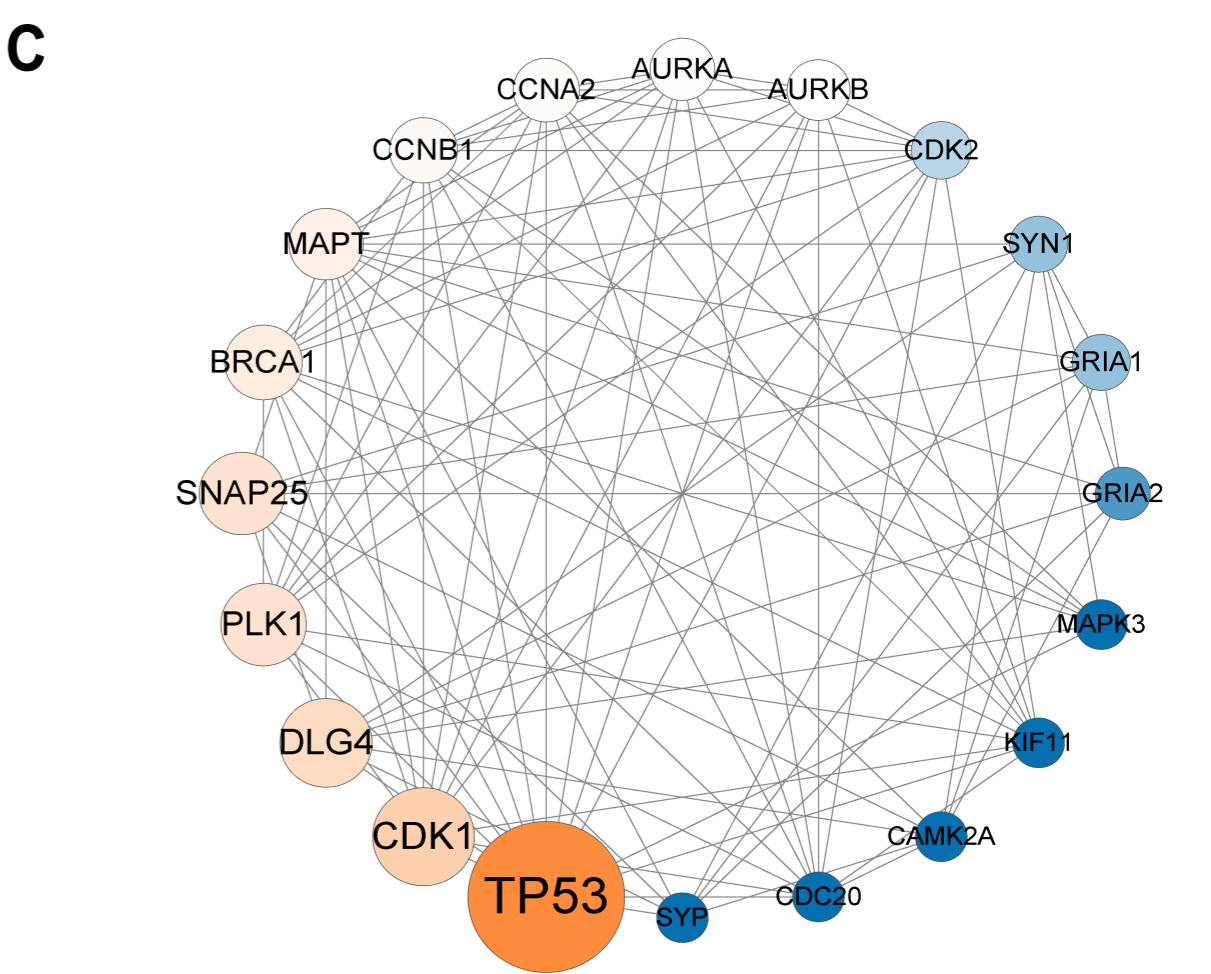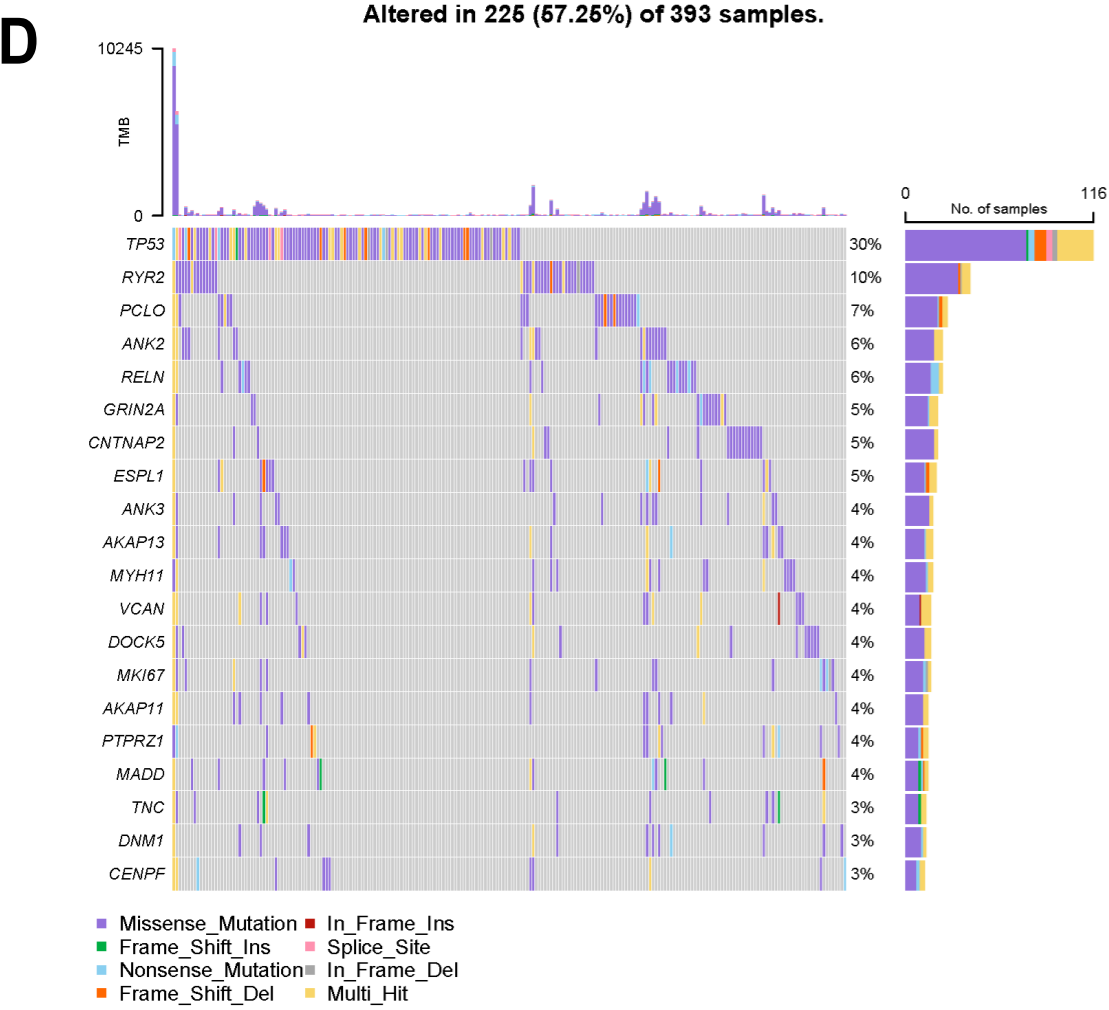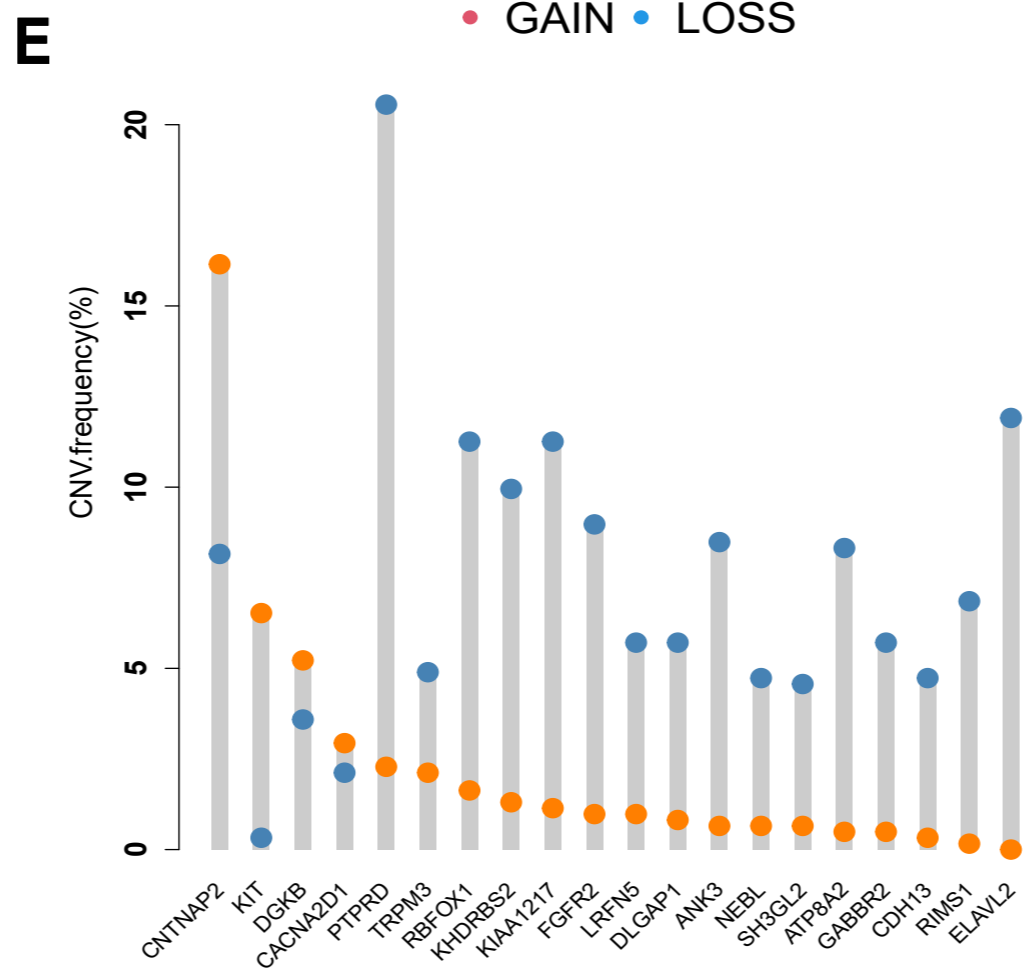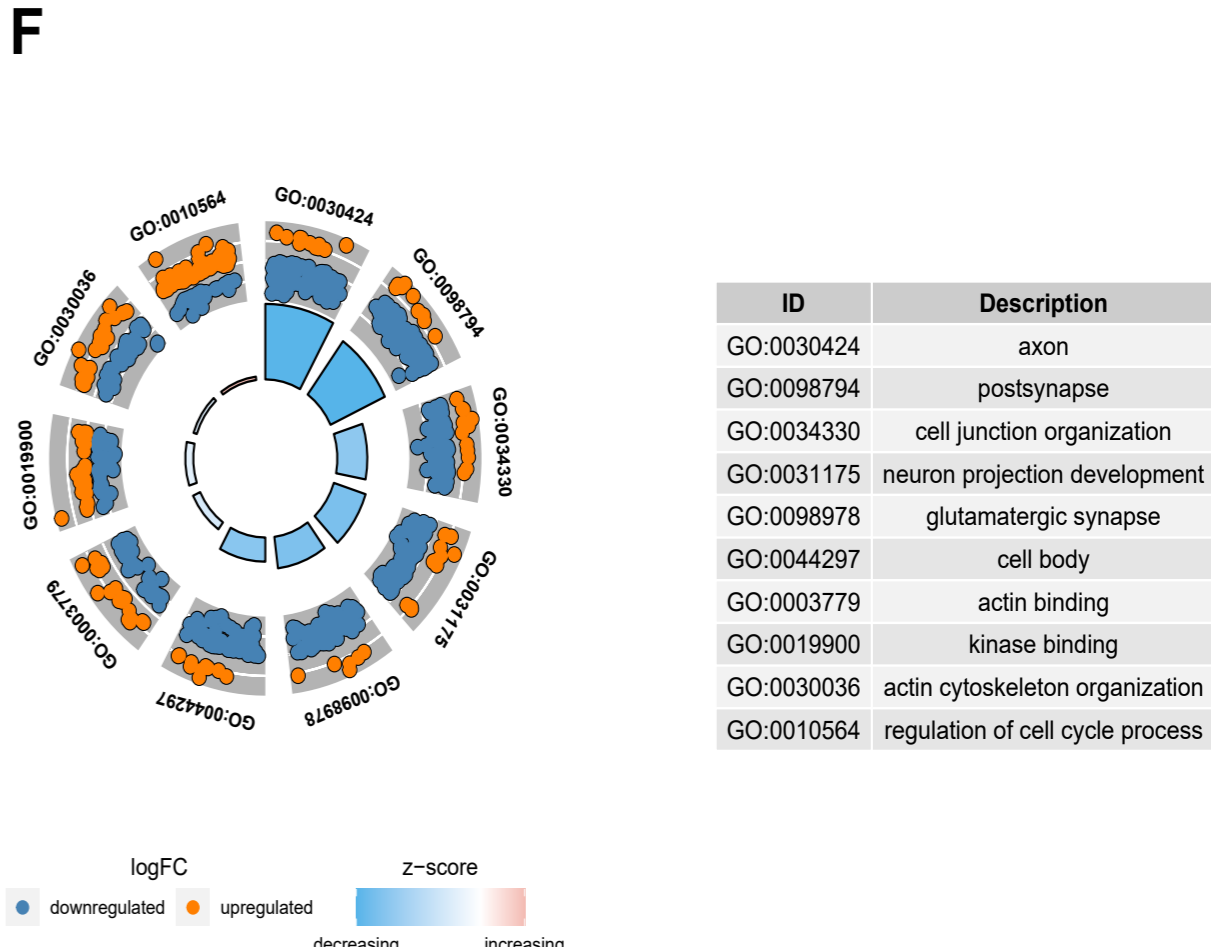

Supplement: Multimedia component 7 [file mmc7.pdf]

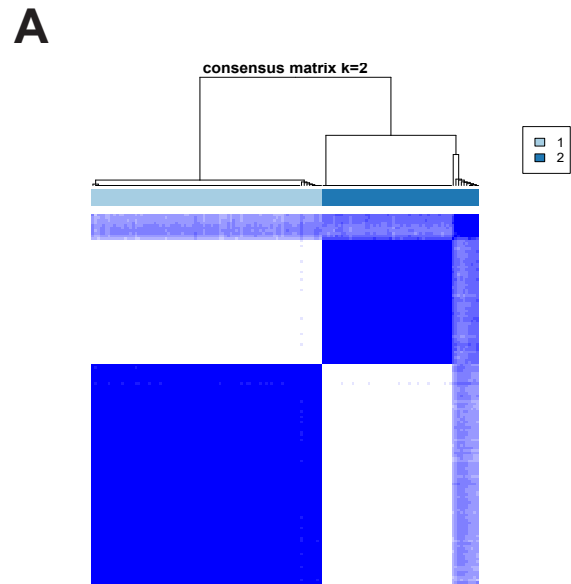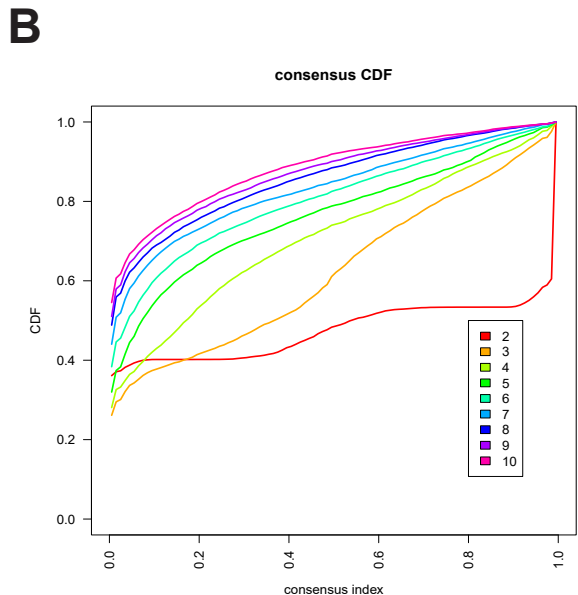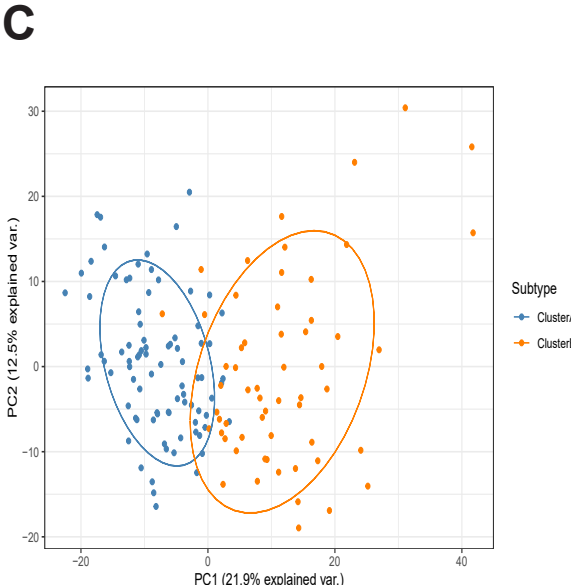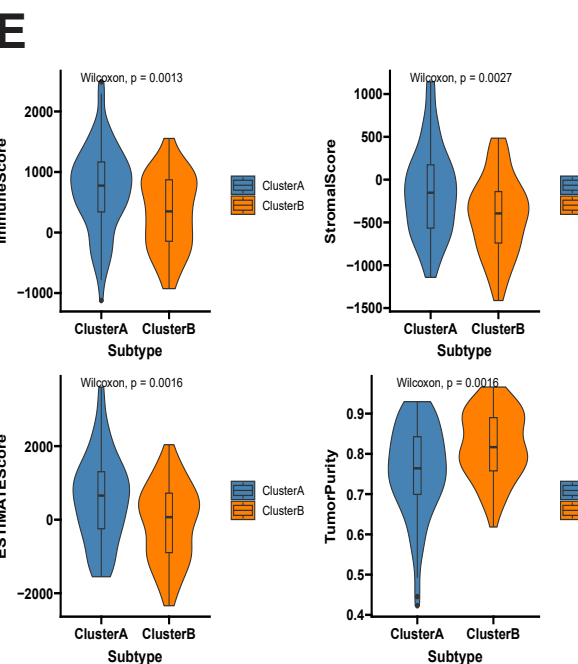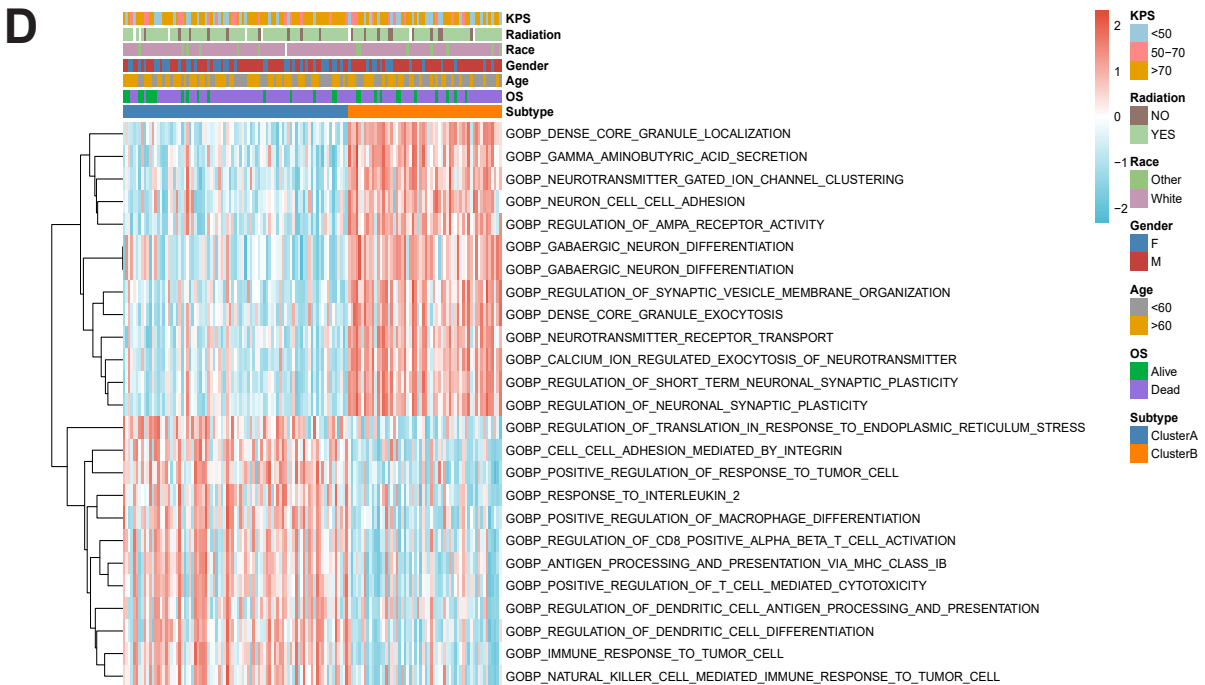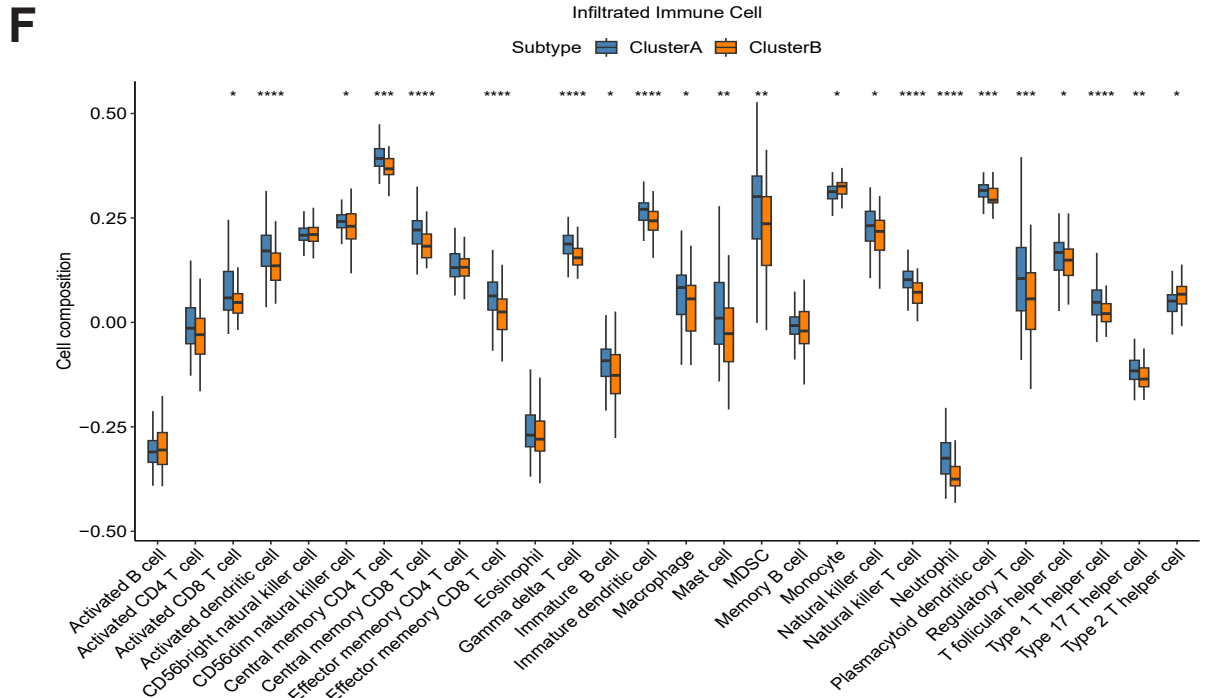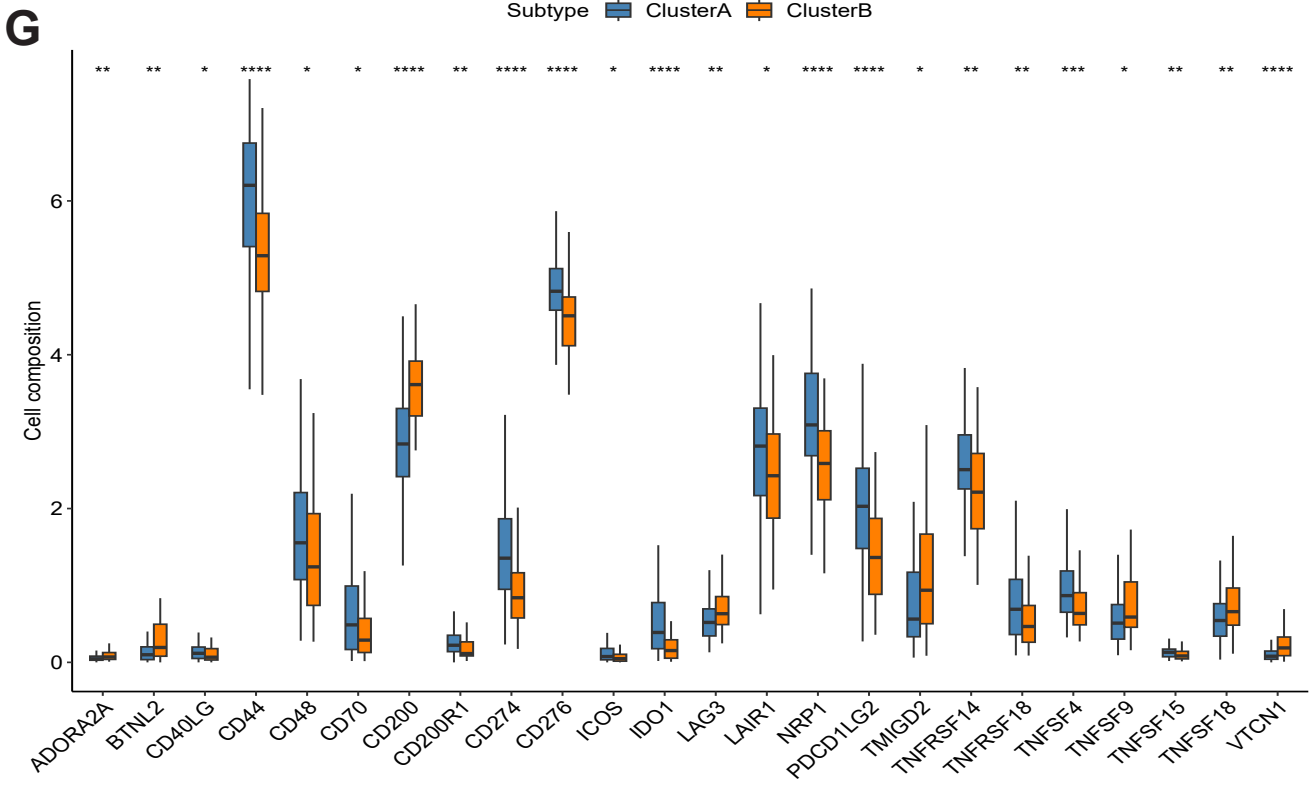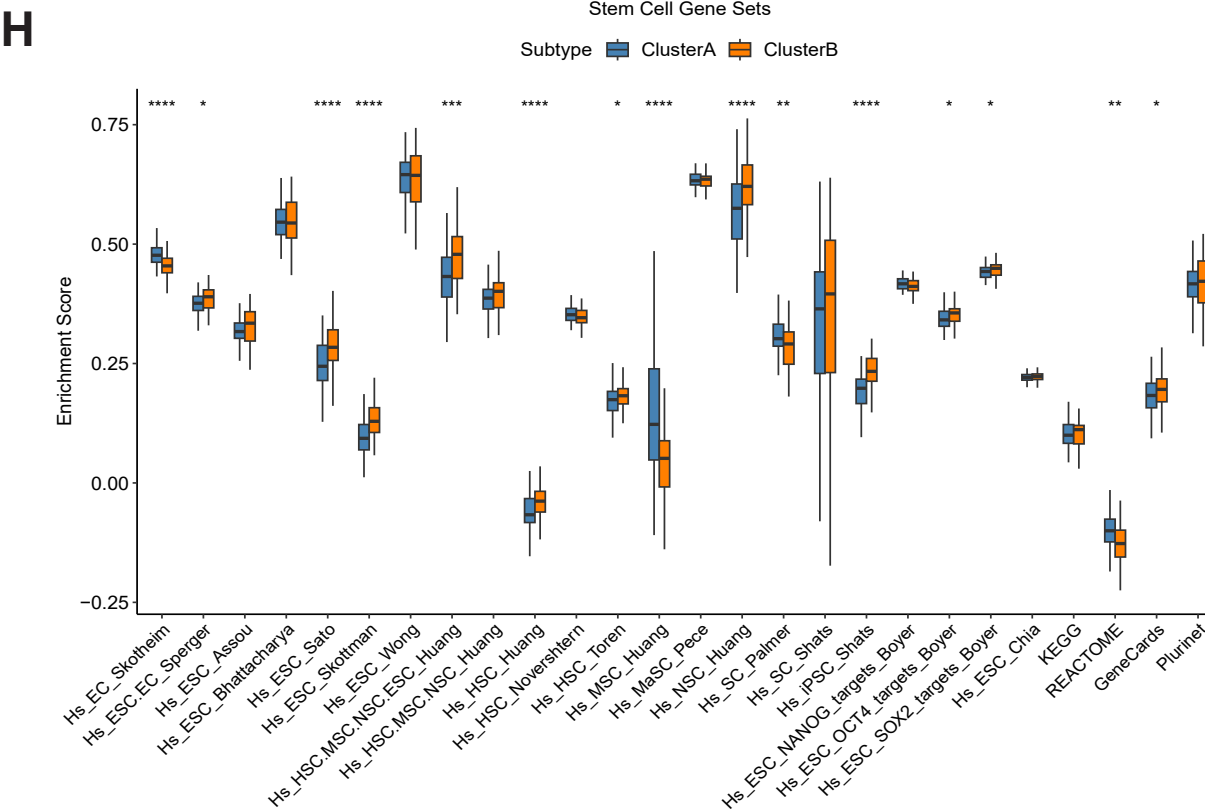

Supplement: Multimedia component 8 [file mmc8.pdf]

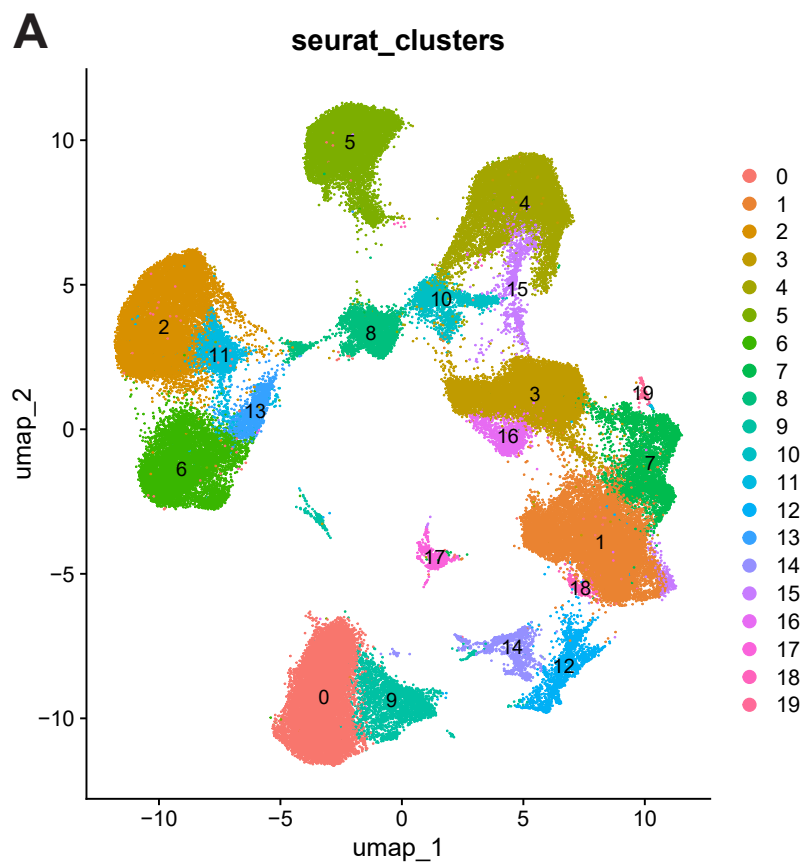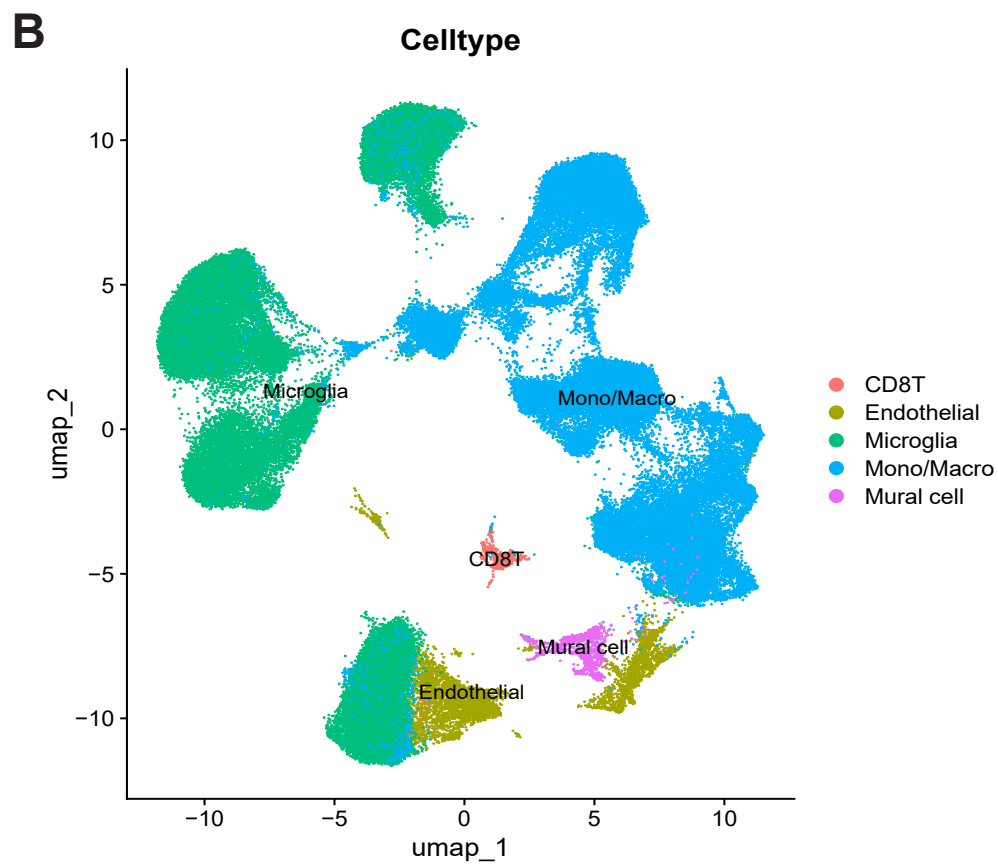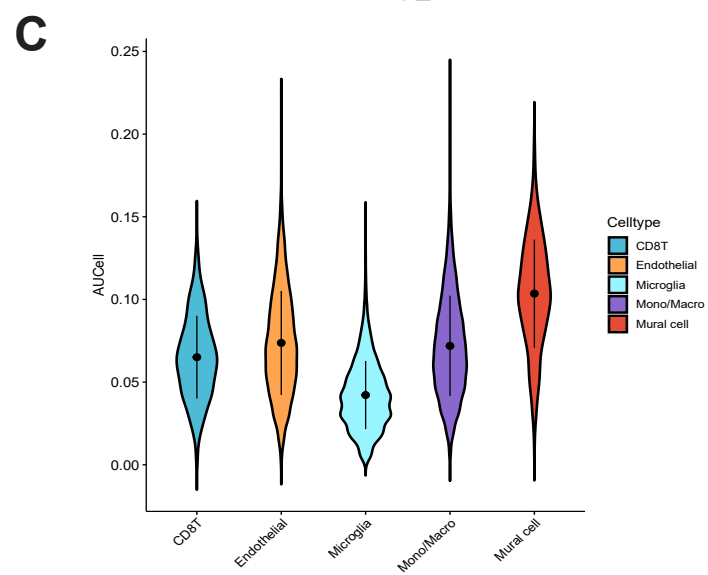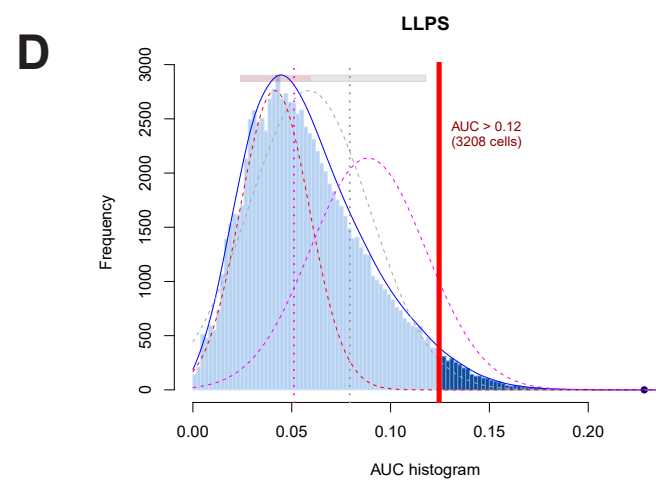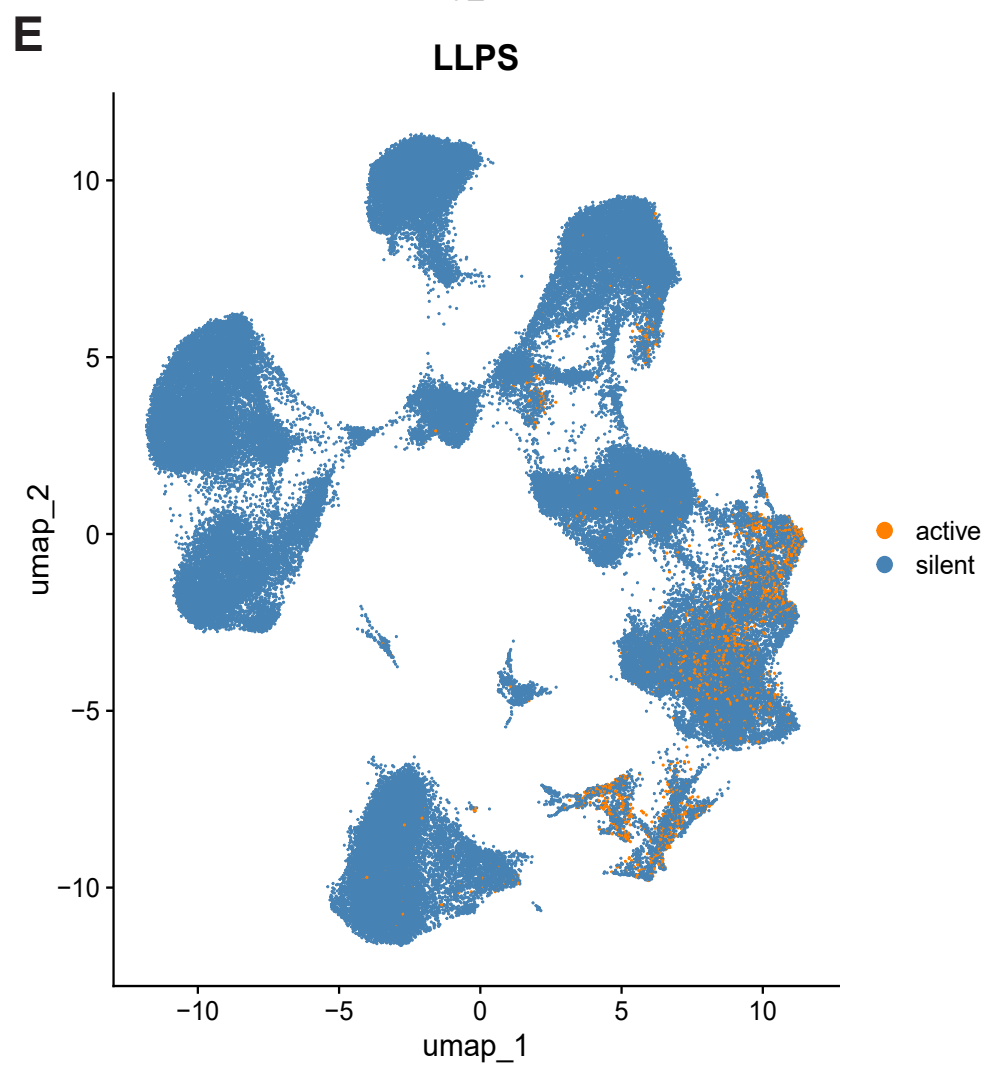

Supplement: Multimedia component 9 [file mmc9.pdf]

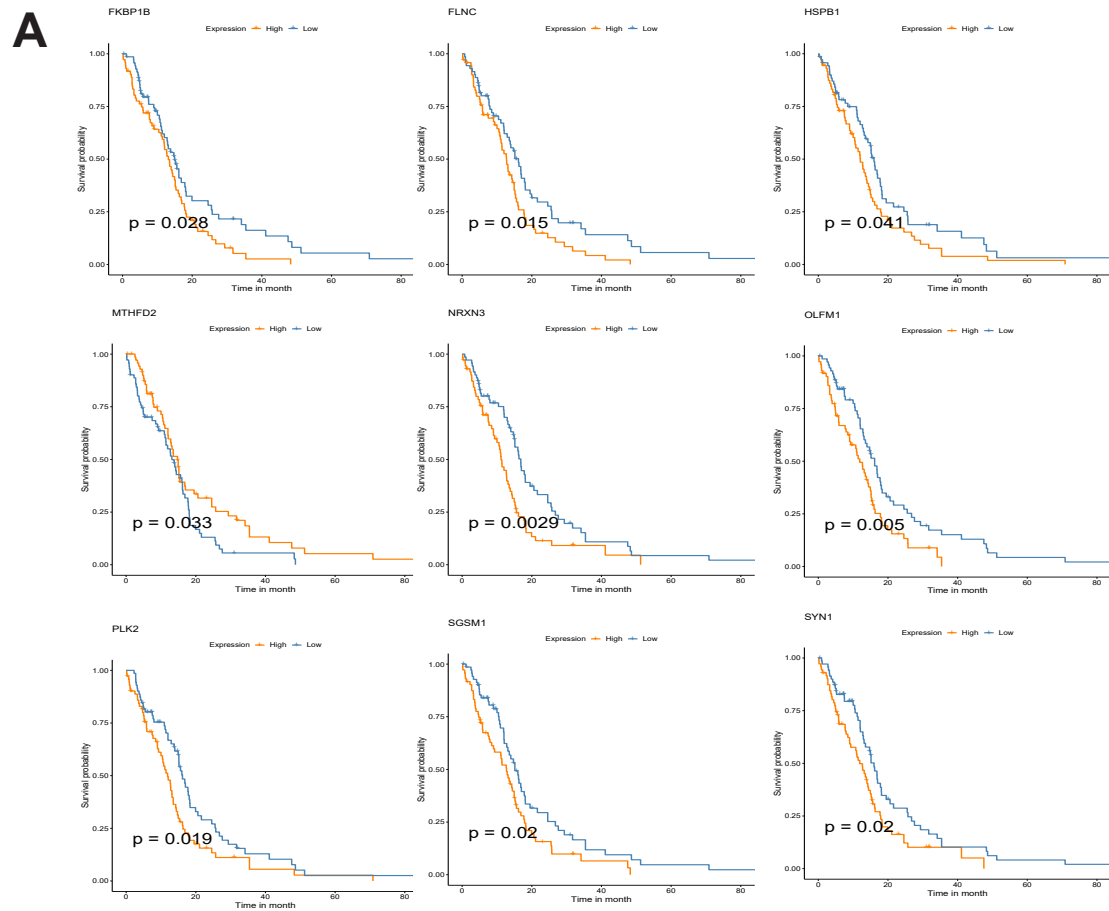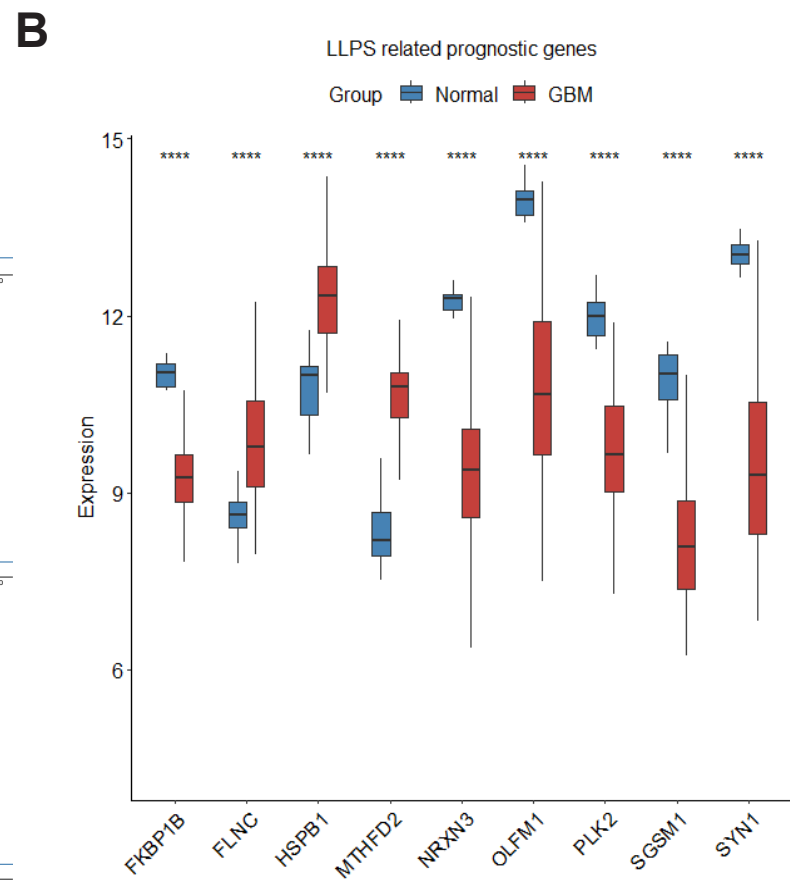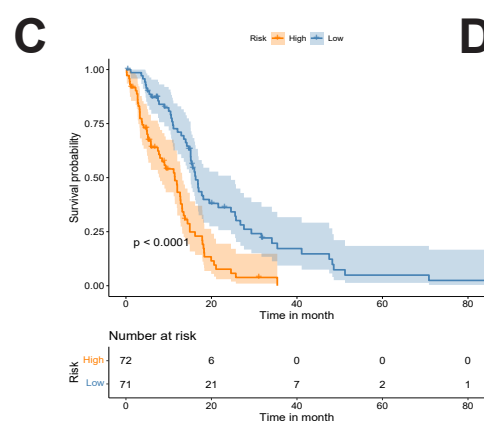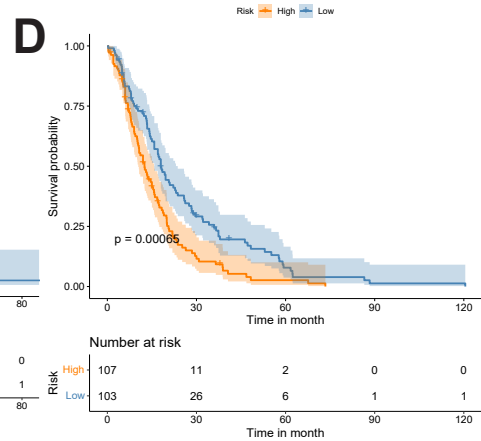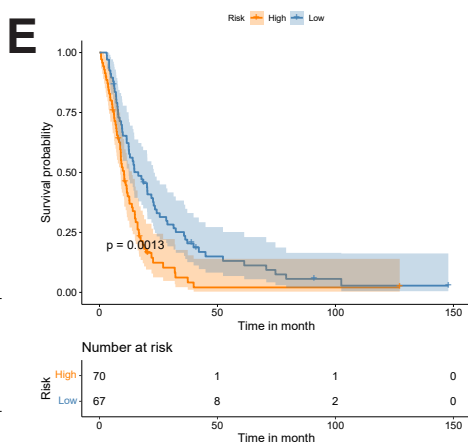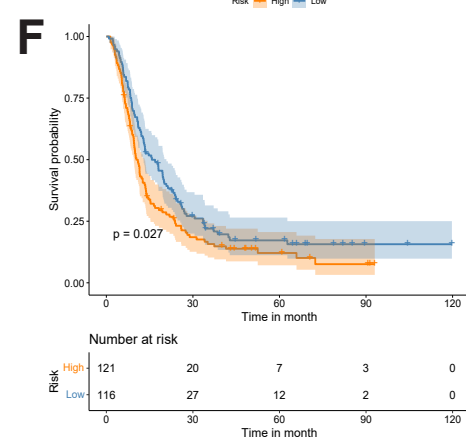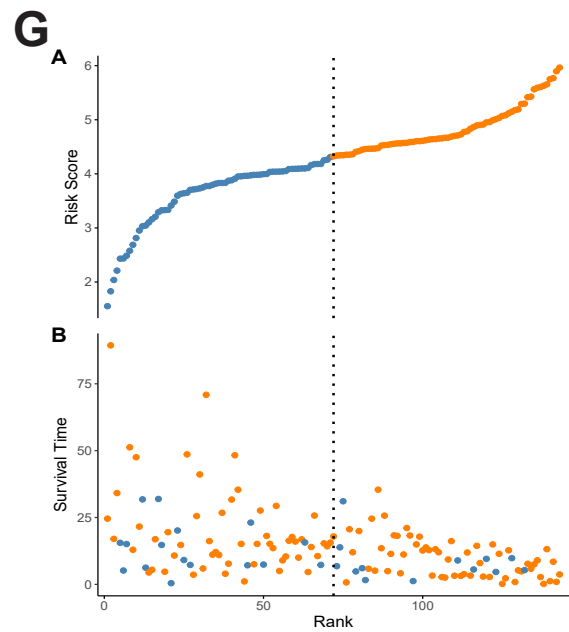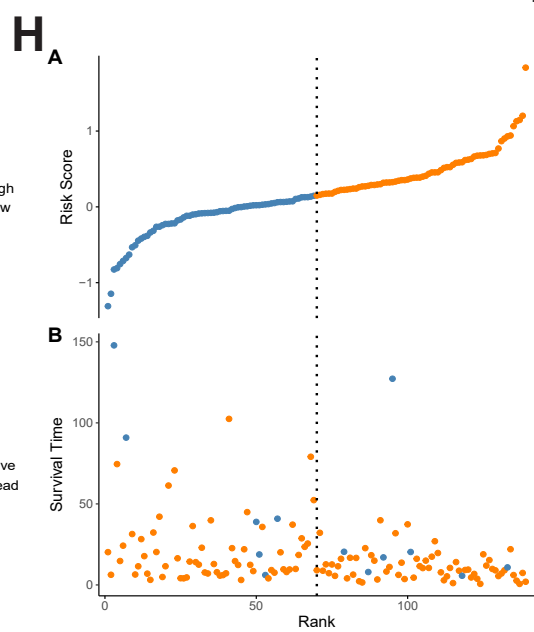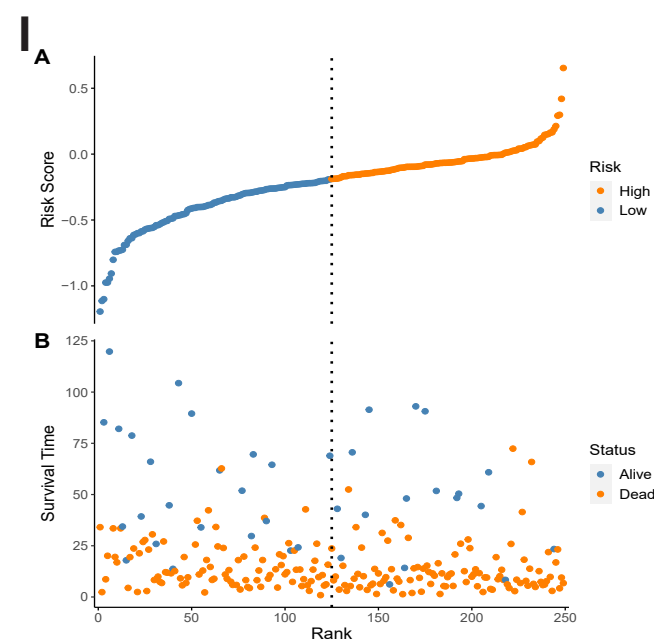

Supplement: Multimedia component 10 [file mmc10.pdf]

A

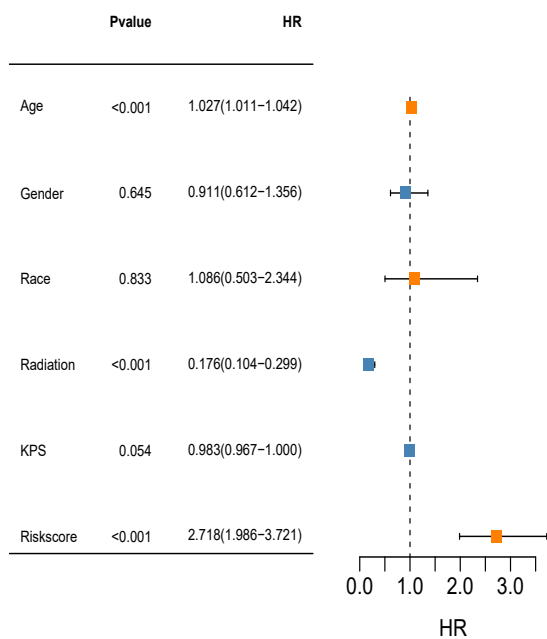

B

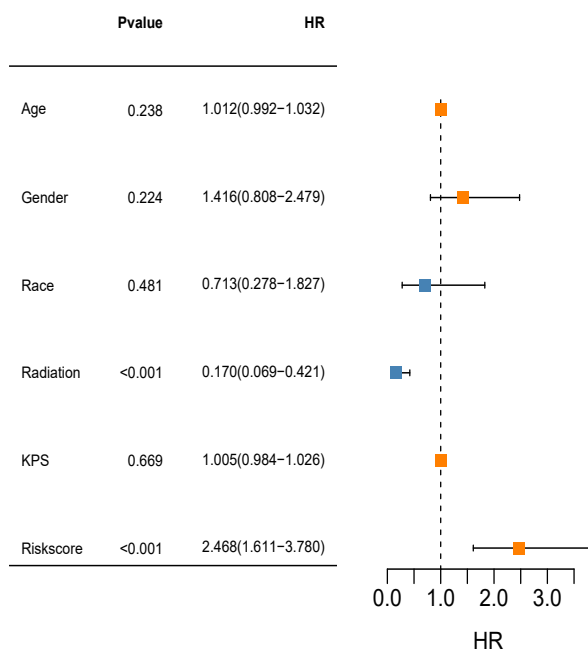

C

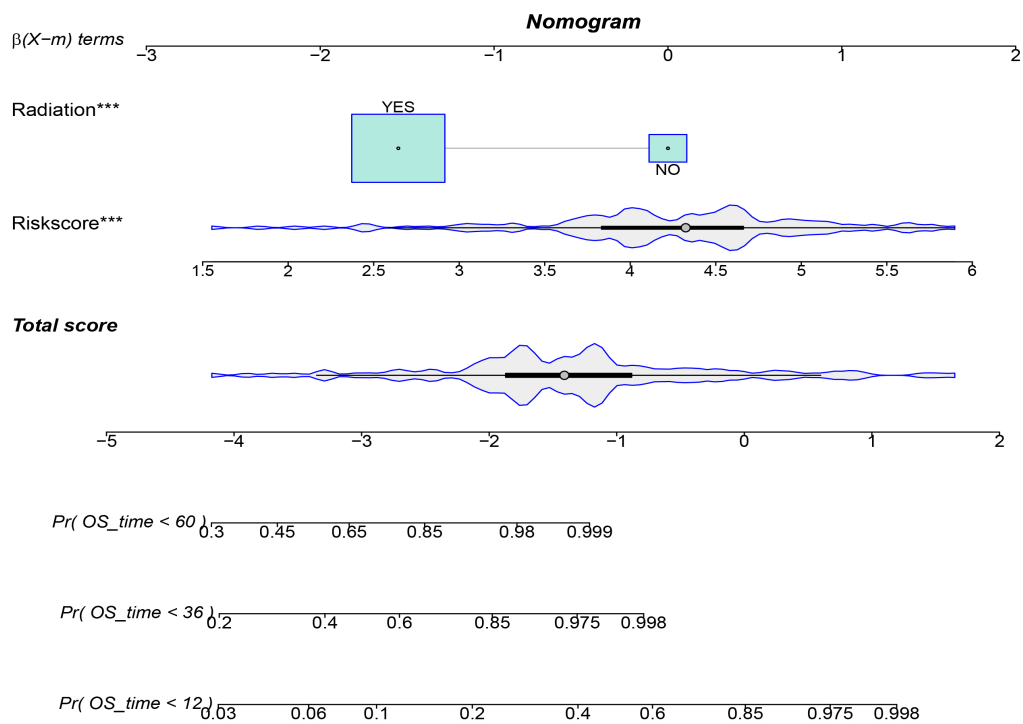

D

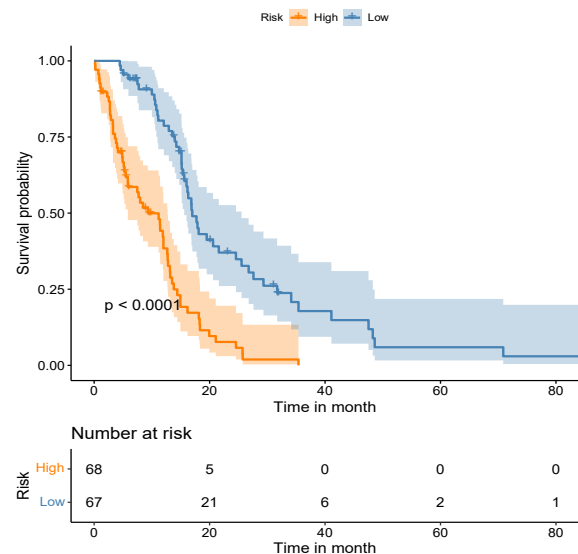

E

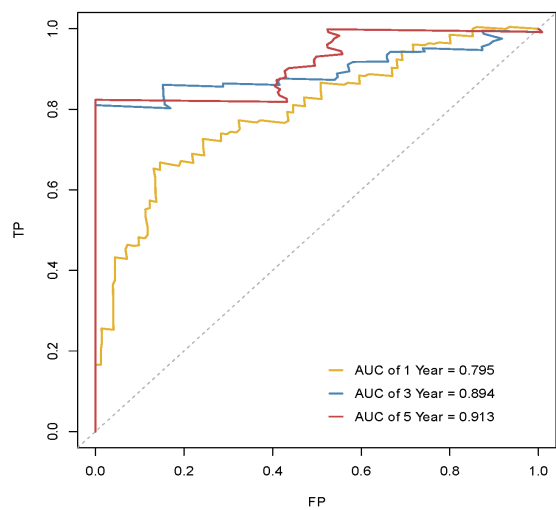

F

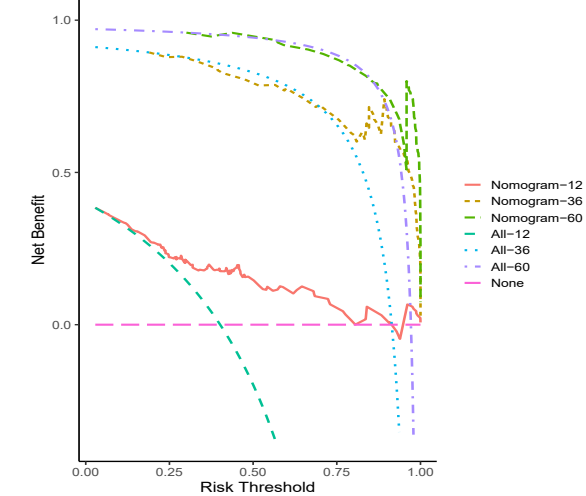

Supplement: Multimedia component 11 [file mmc11.pdf]

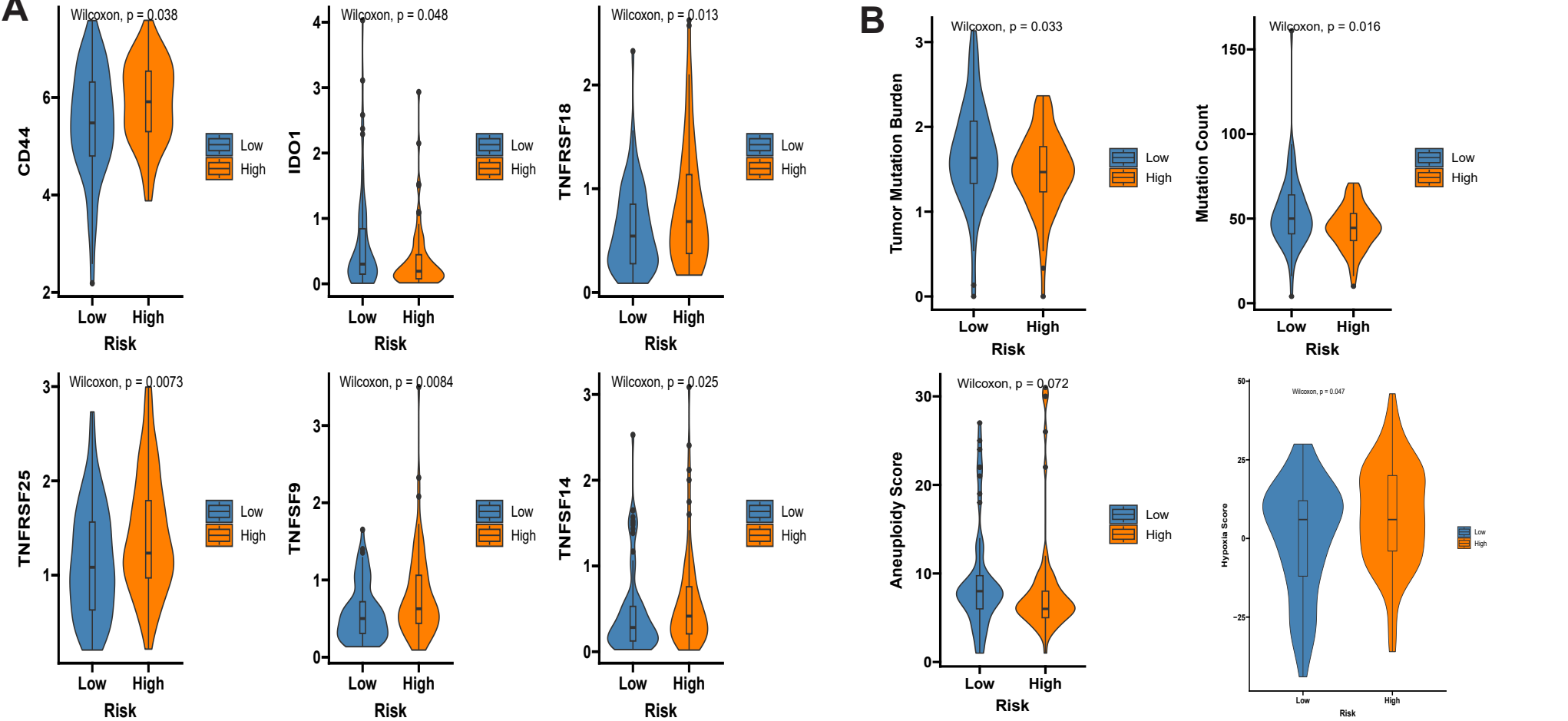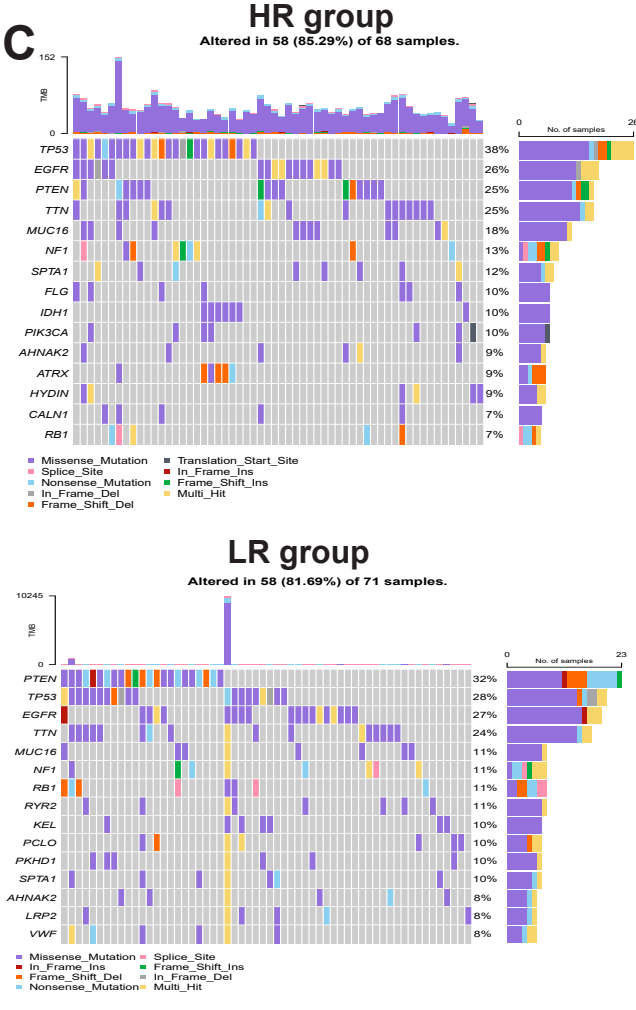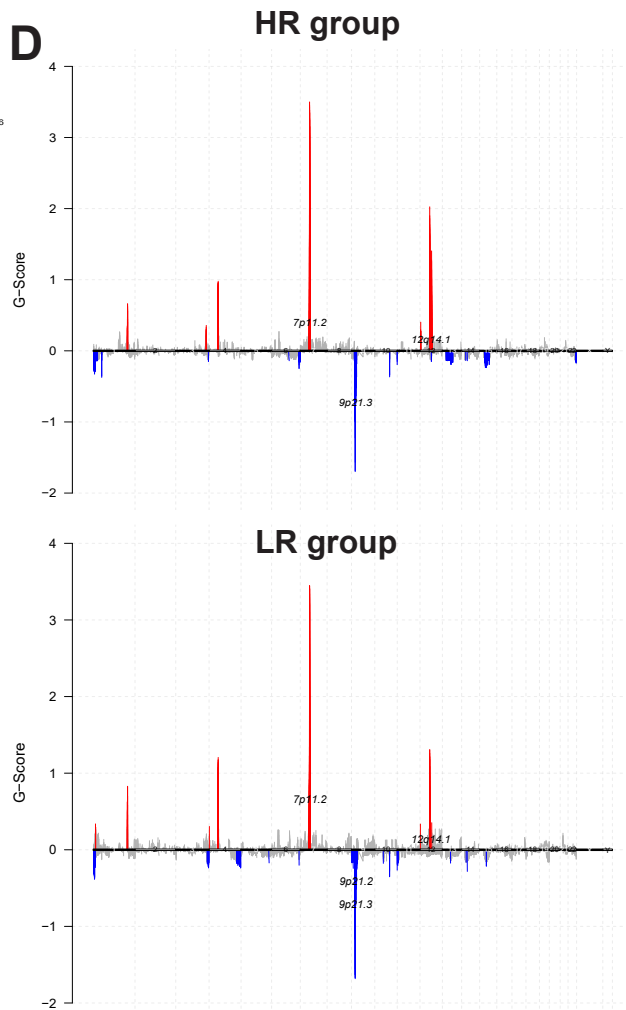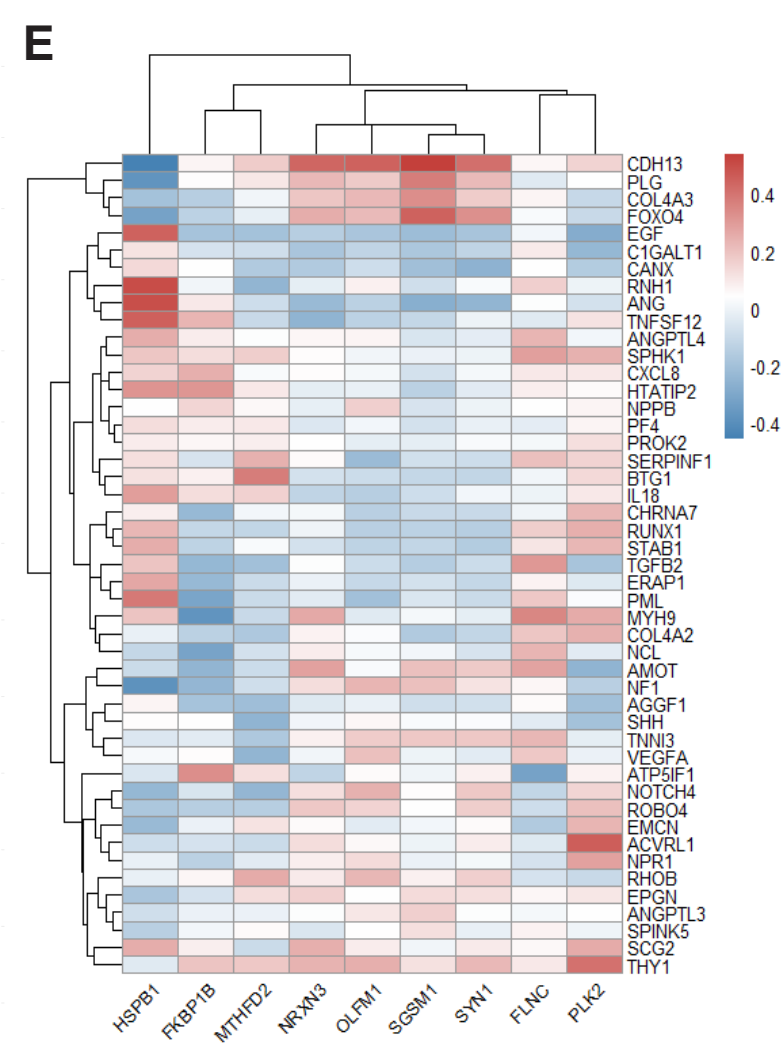

Supplement: Multimedia component 12 [file mmc12.pdf]

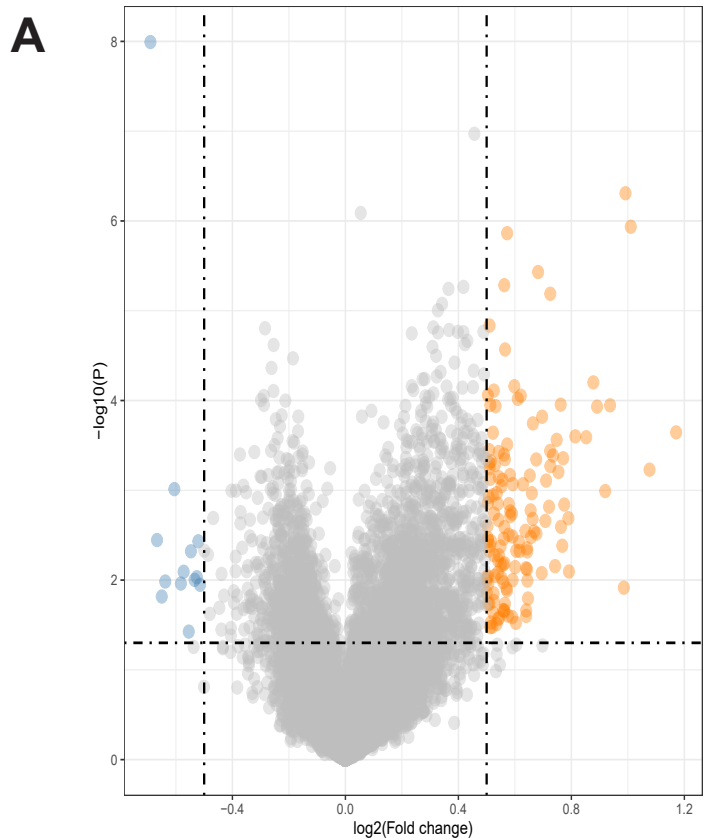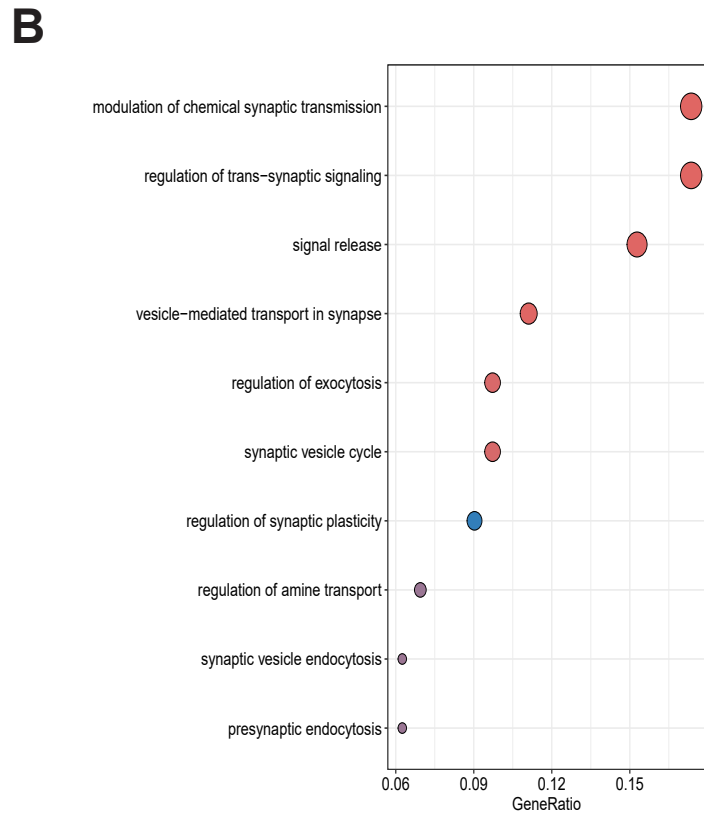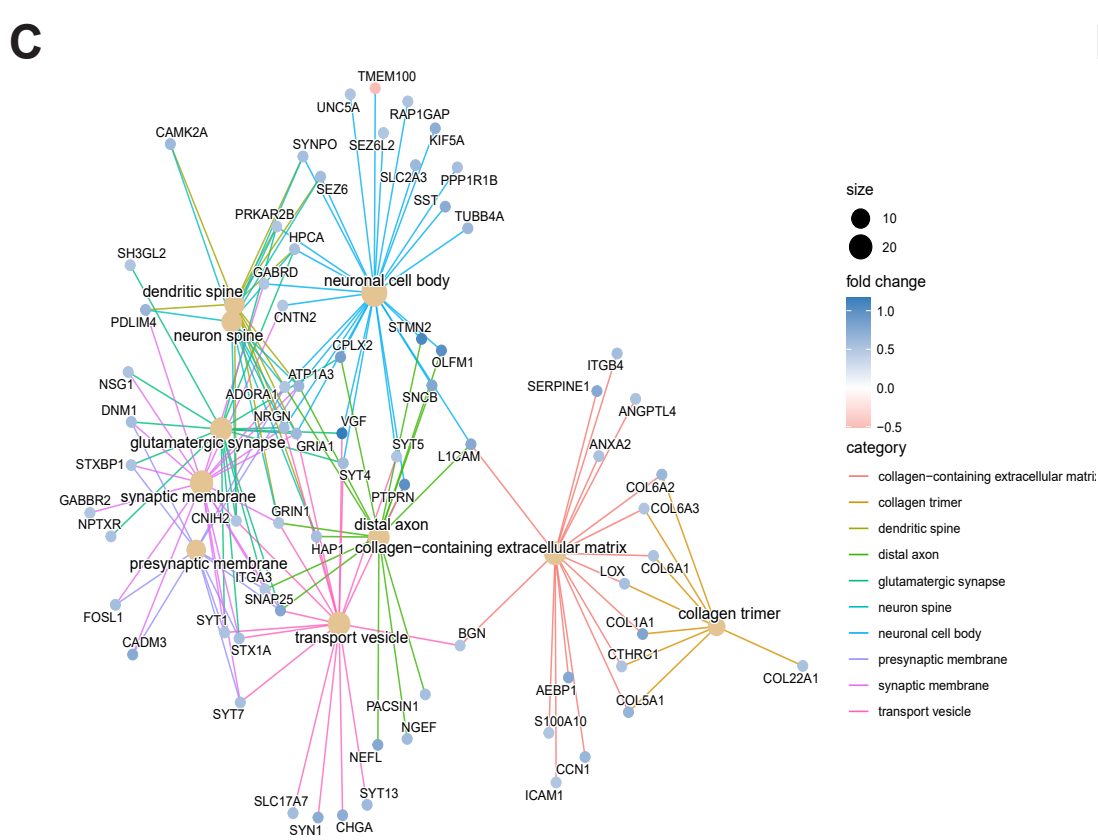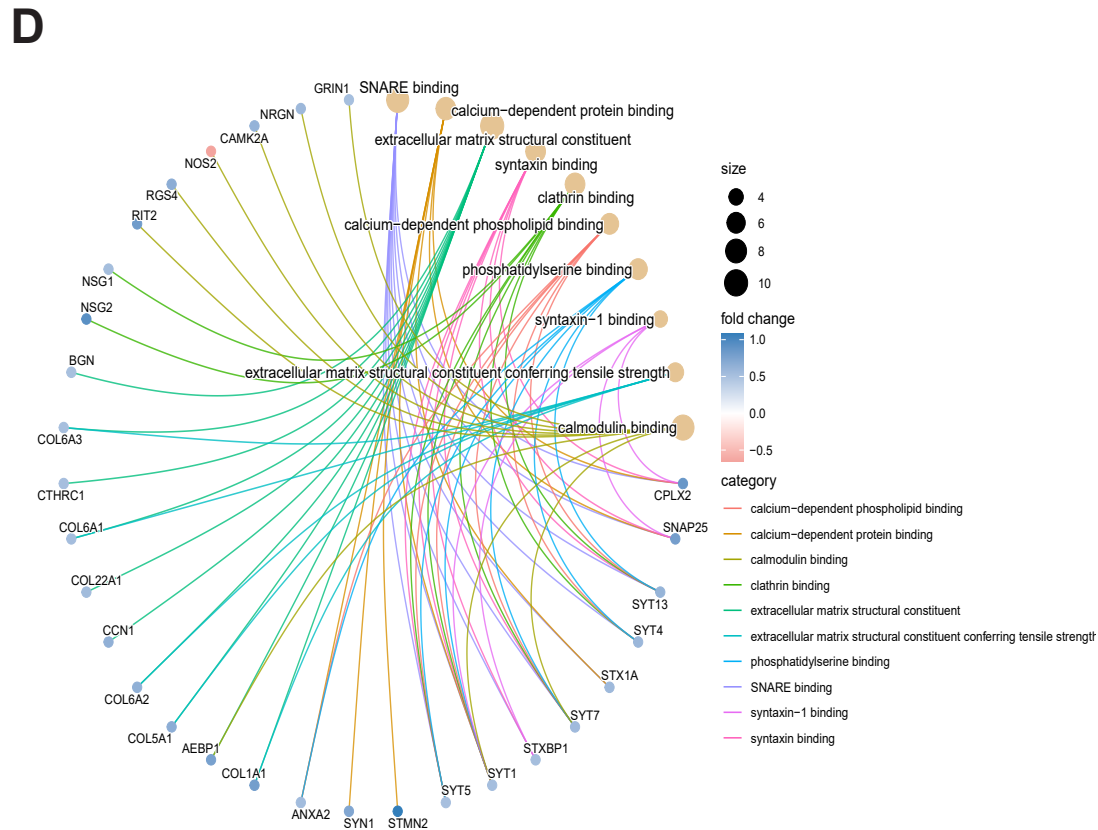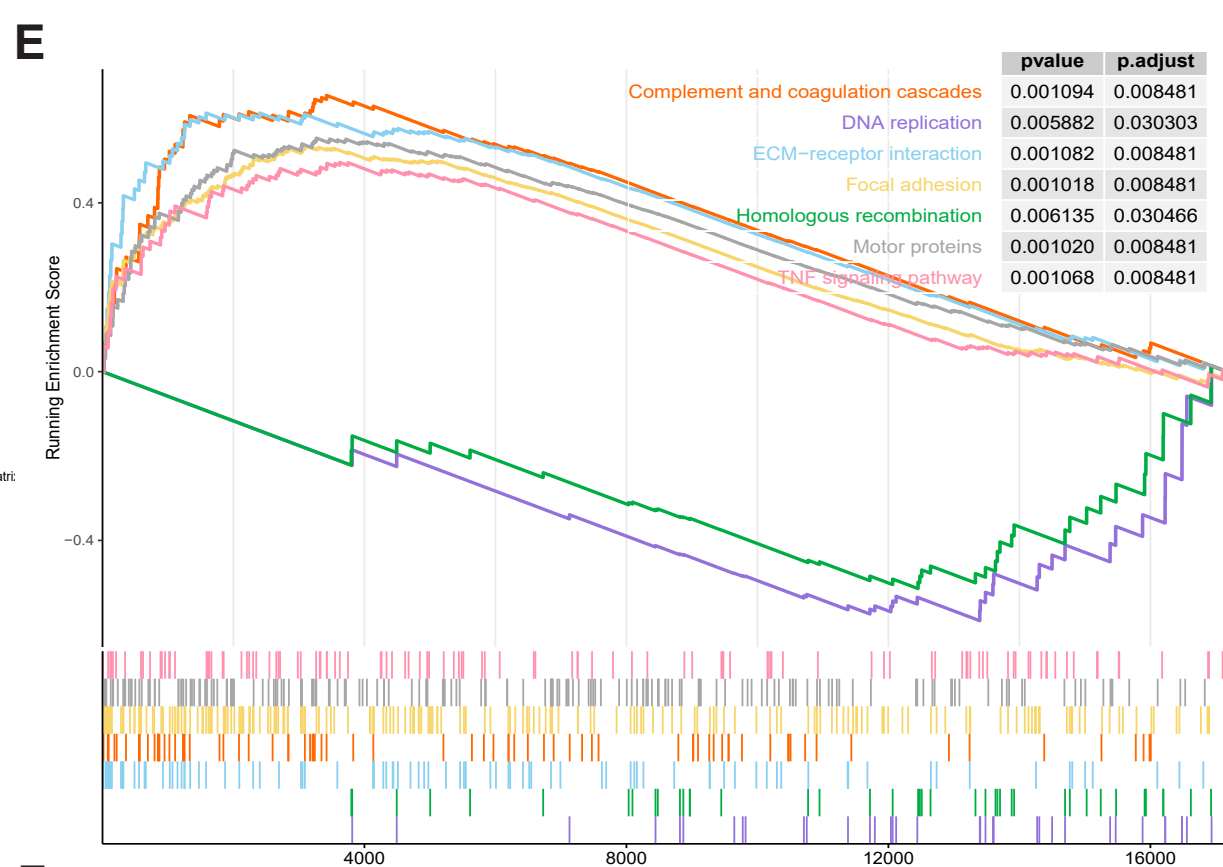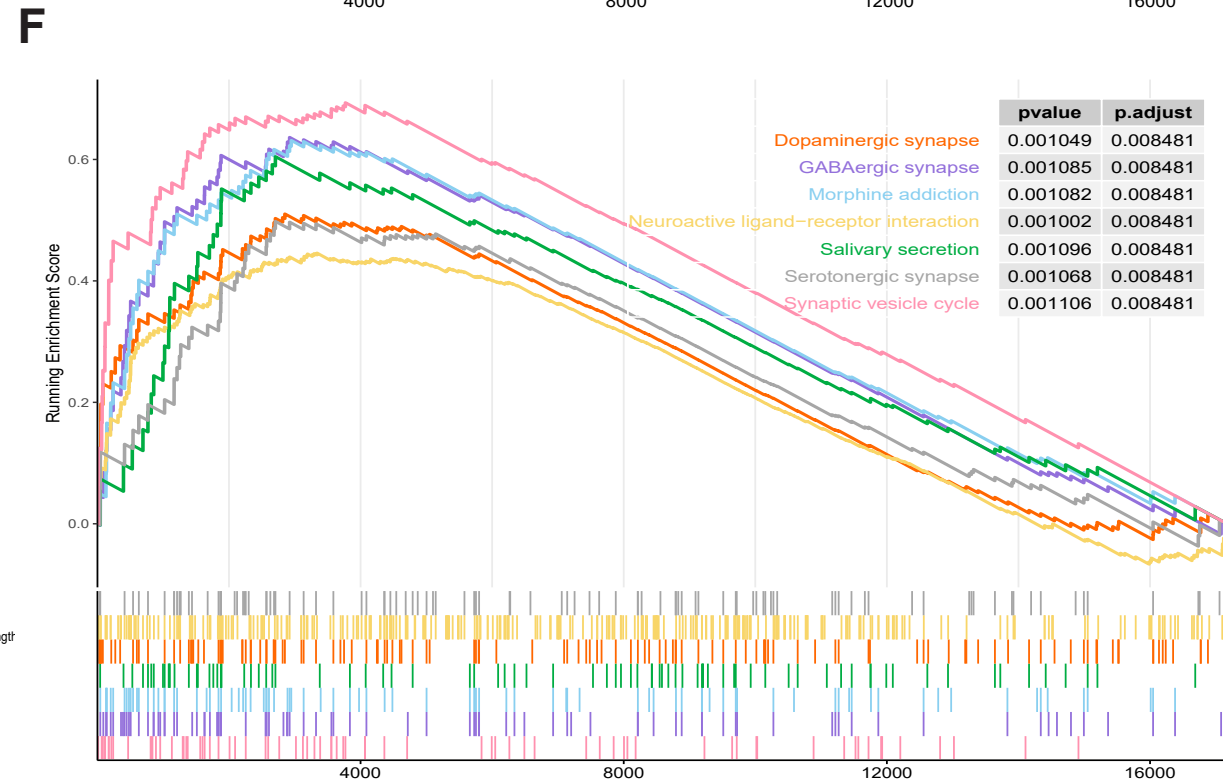

Supplement: Multimedia component 13 [file mmc13.pdf]

# A

## Correlation of marker gene expression with drug sensitivity

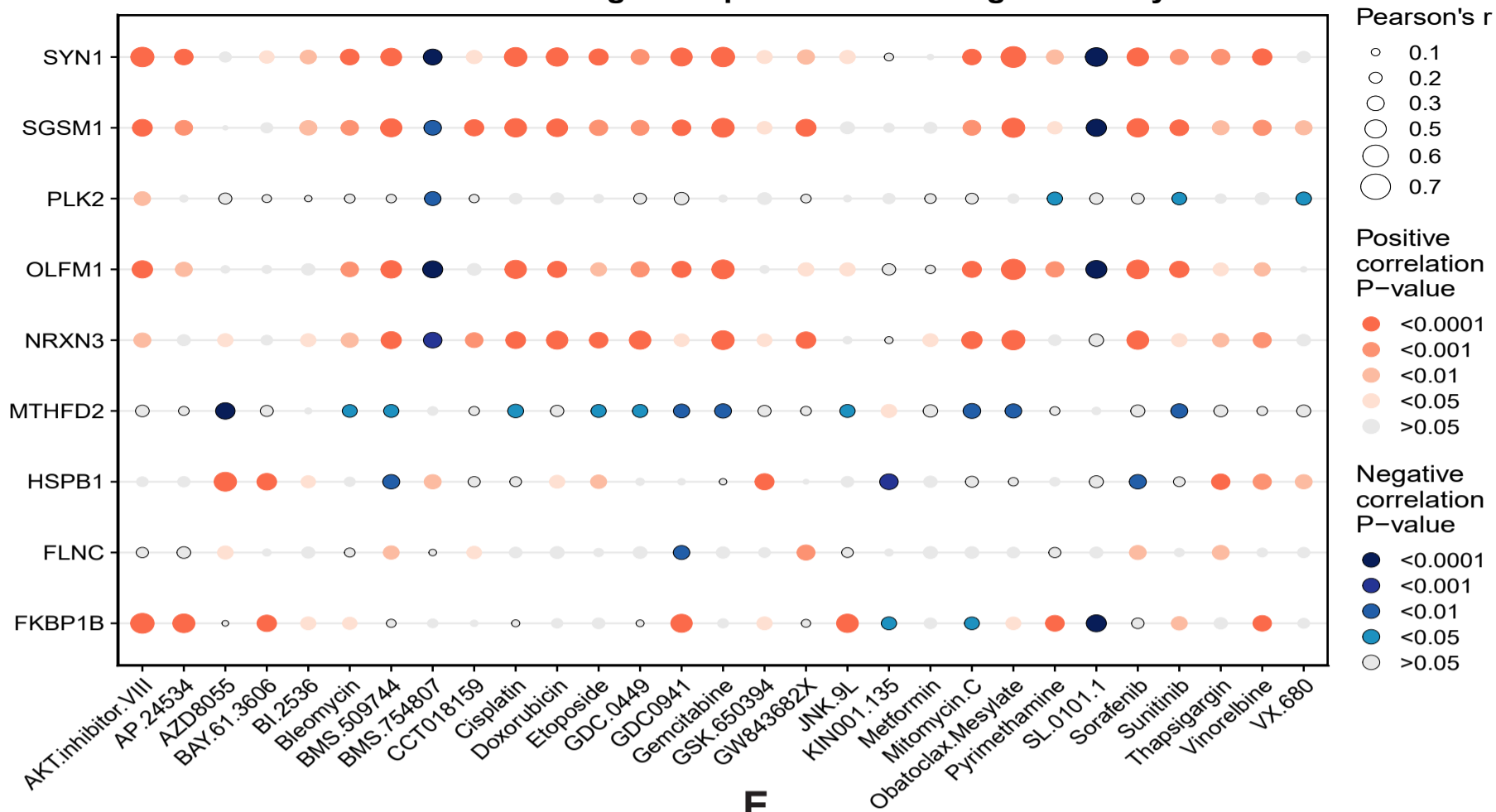

**B**

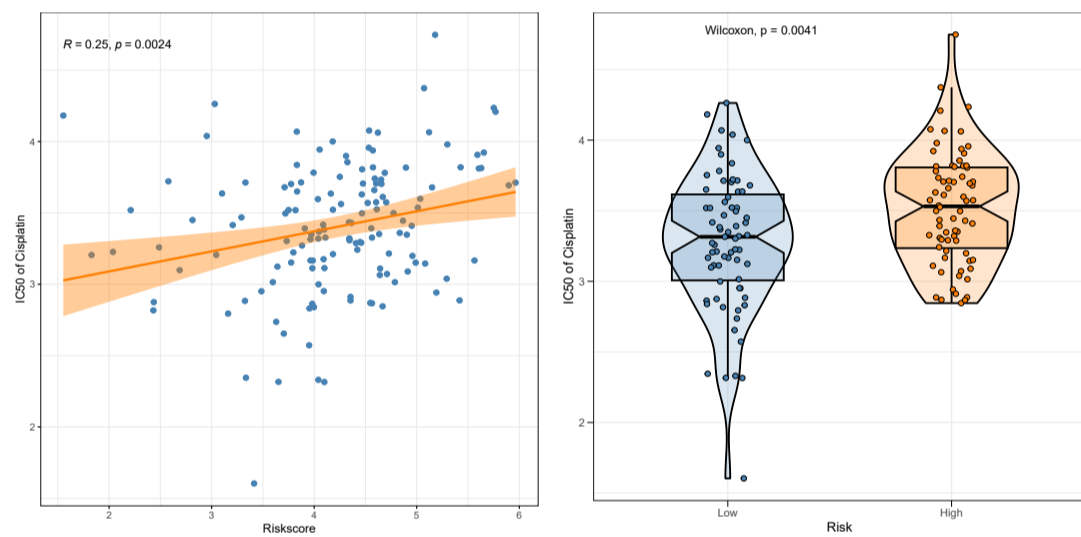

**E**

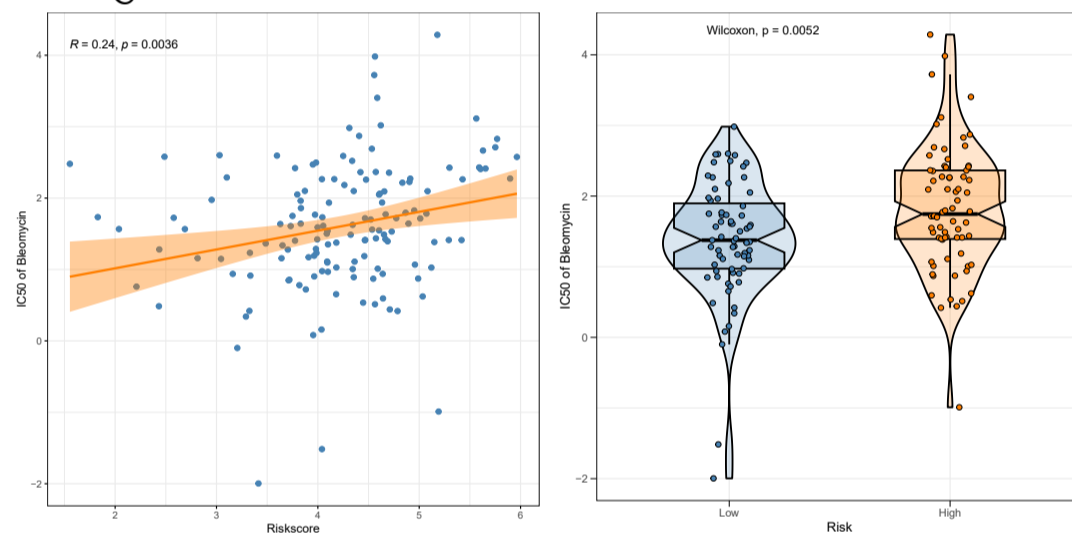

**C**

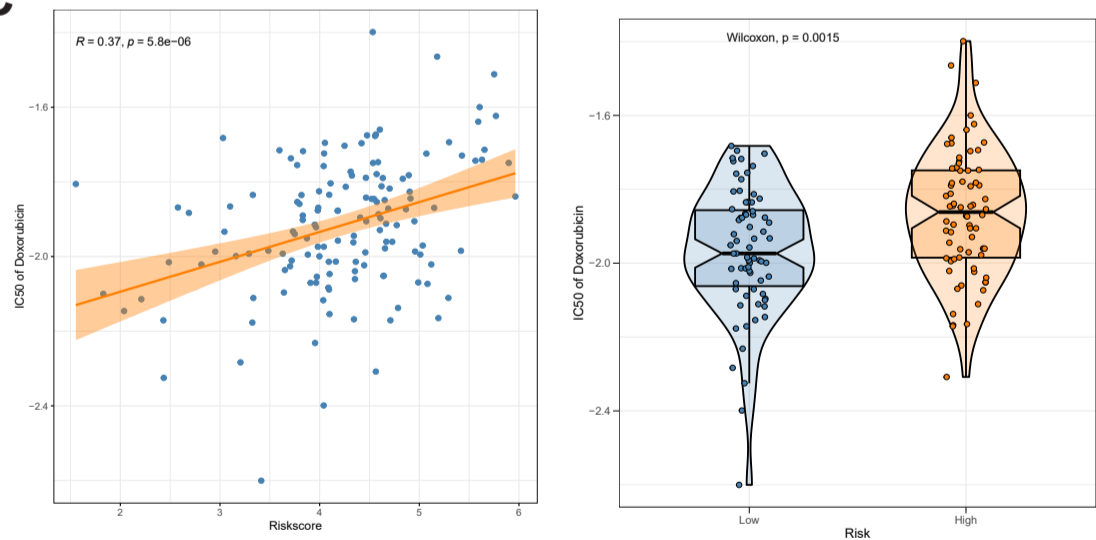

**F**

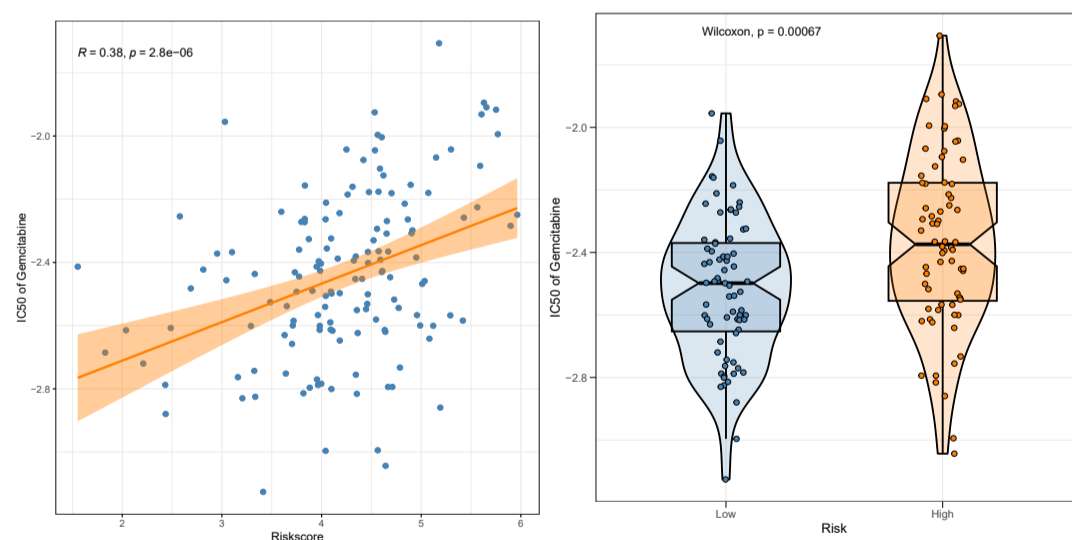

**D**

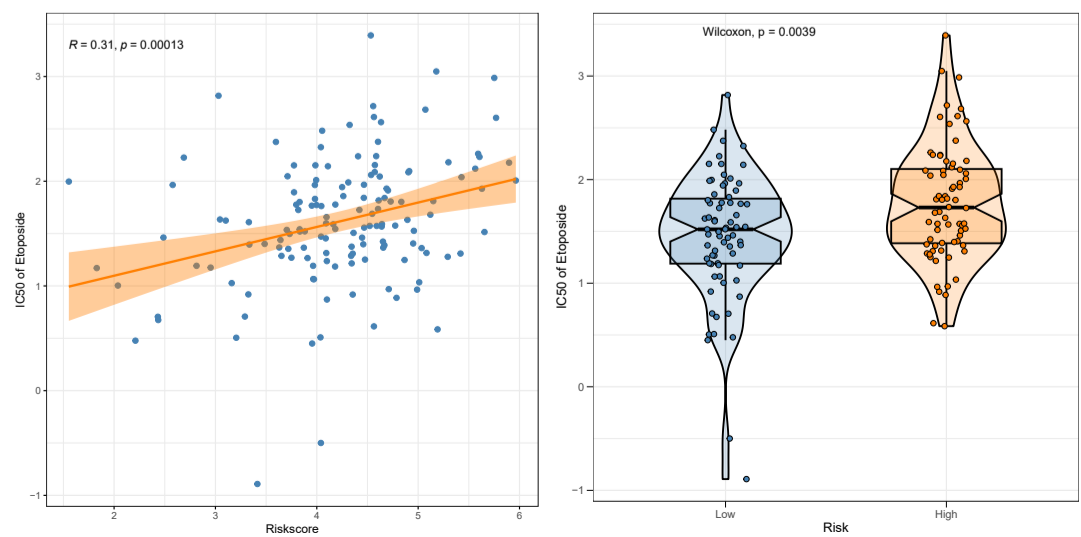

**G**

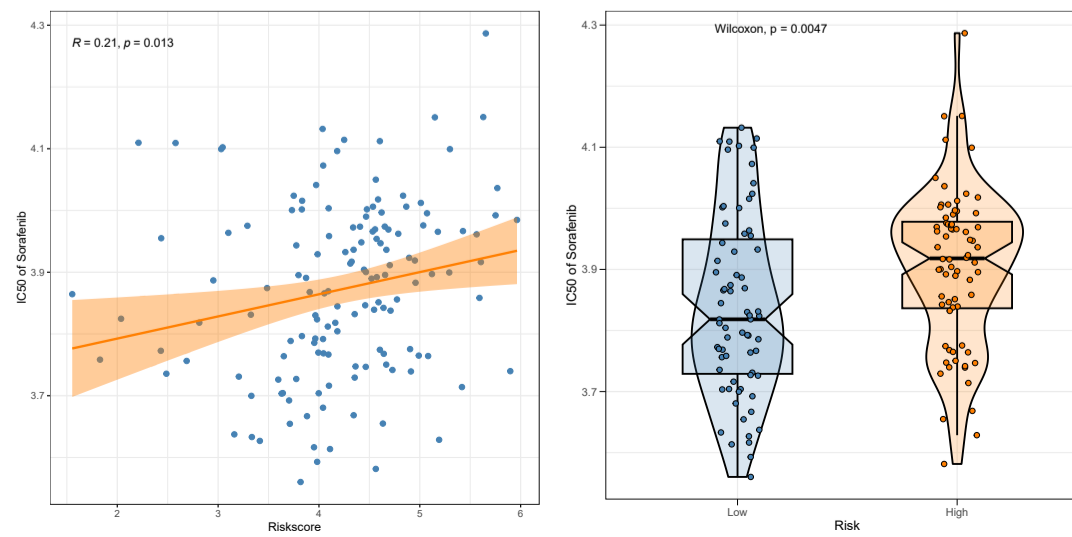

Supplement: Multimedia component 14 [file mmc14.pdf]

**A**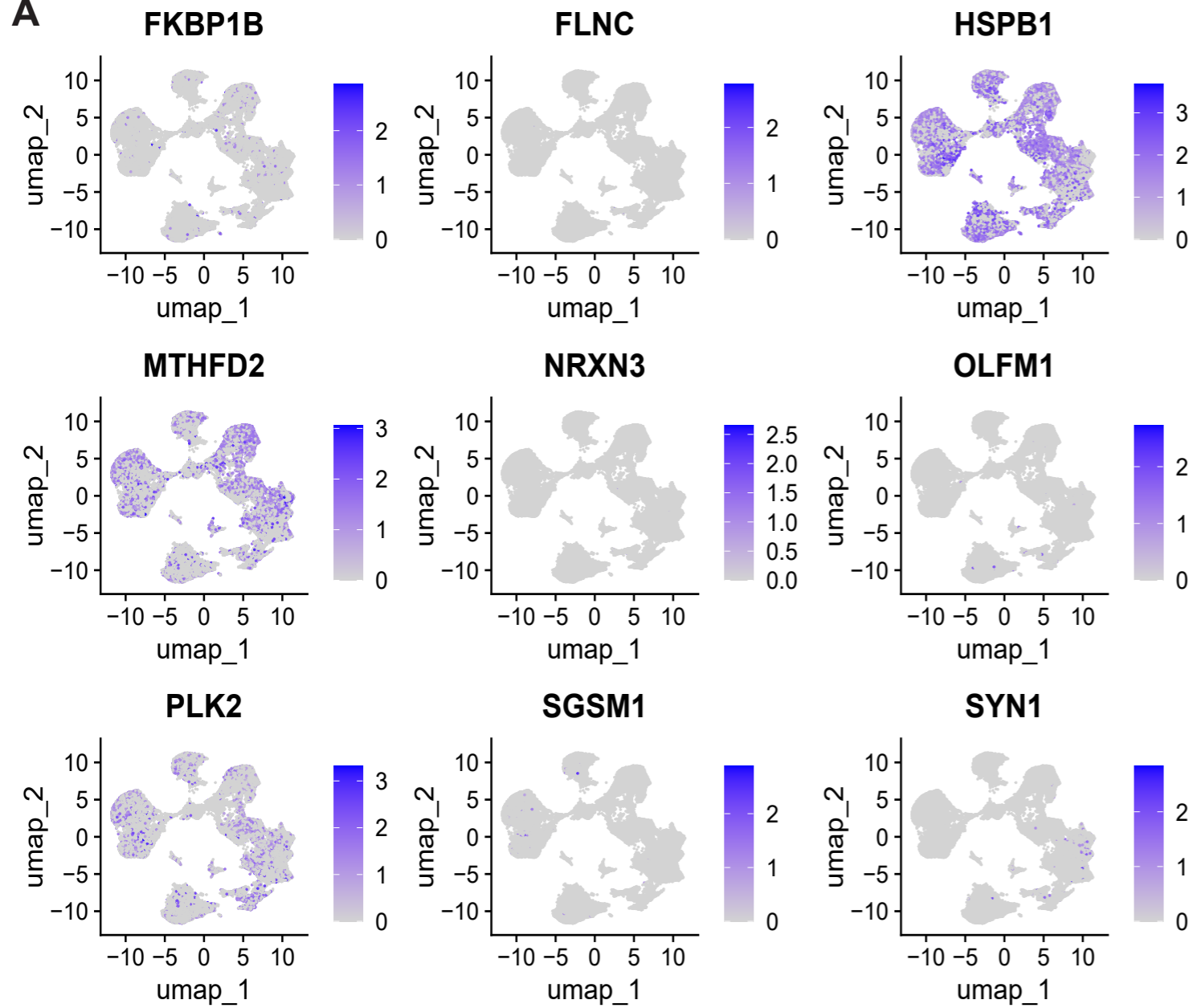**B**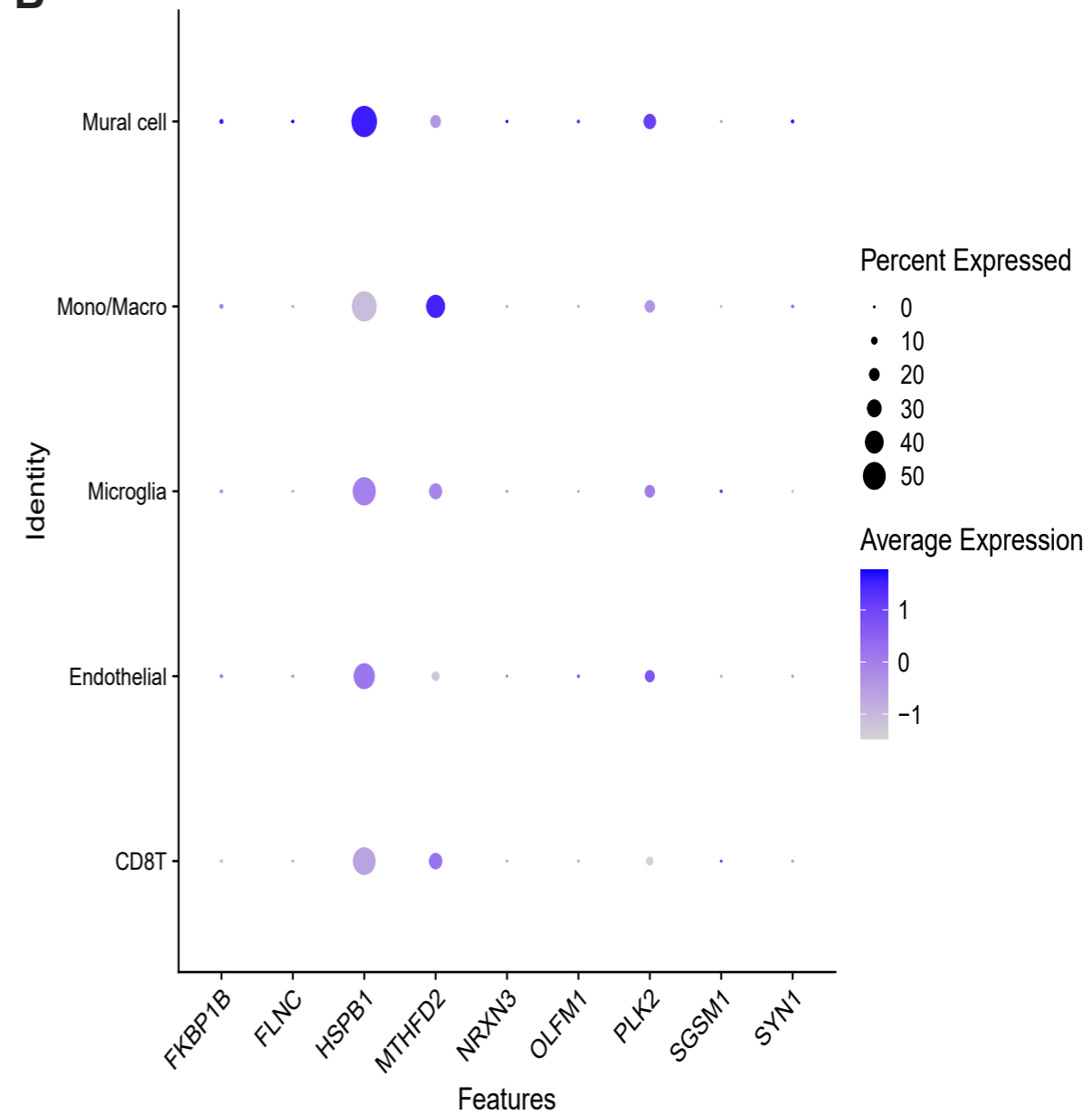

Supplement: Multimedia component 15 [file mmc15.pdf]

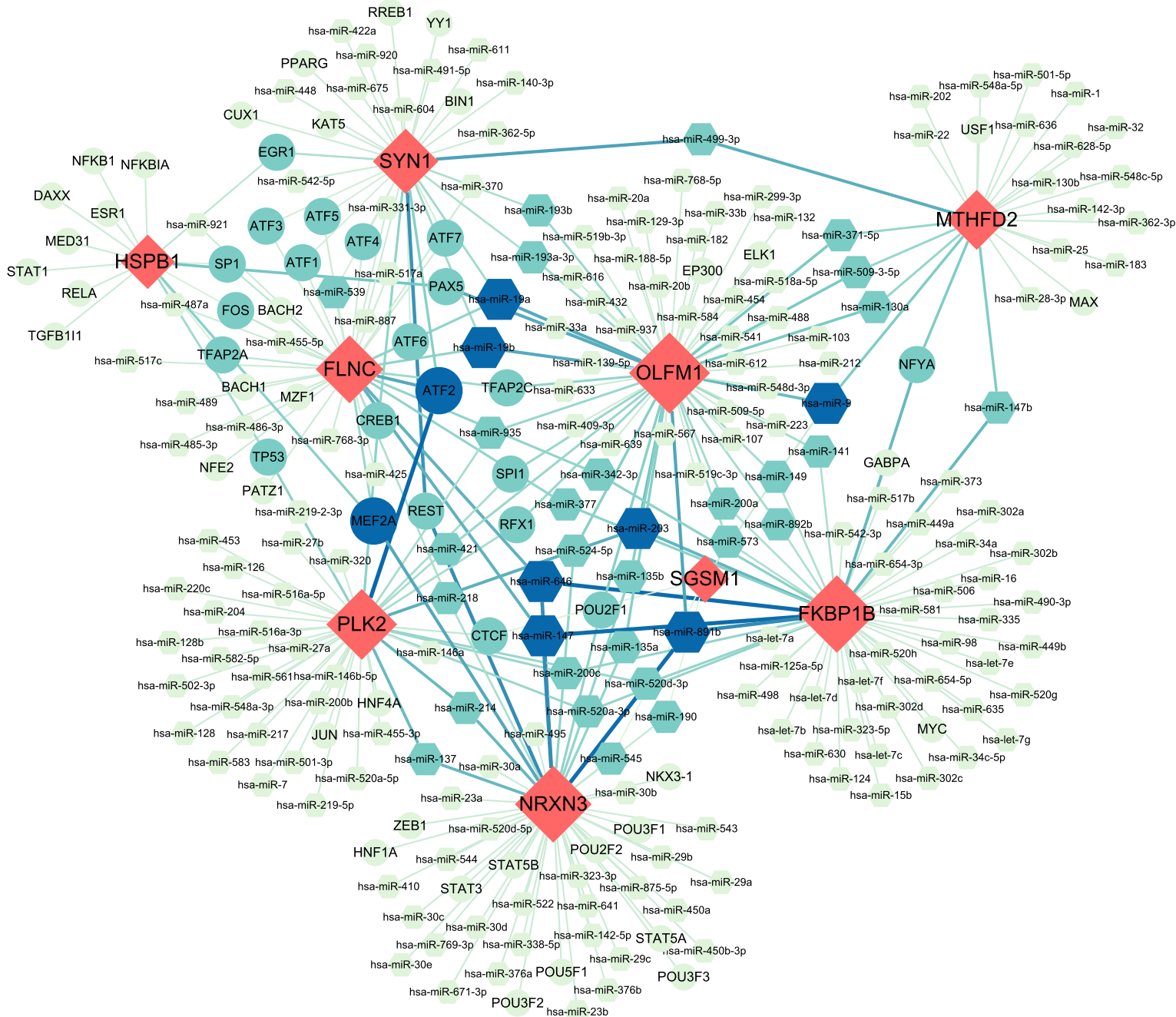

Supplement: Multimedia component 16 [file mmc16.pdf]

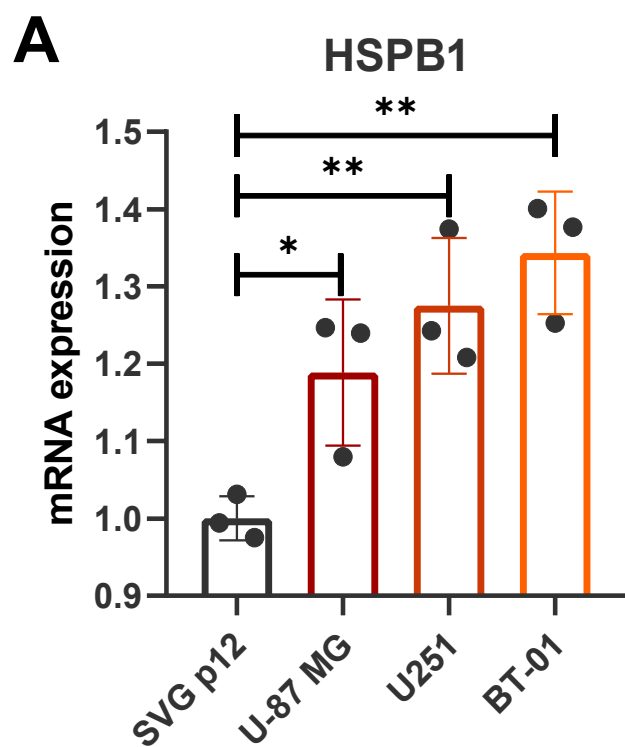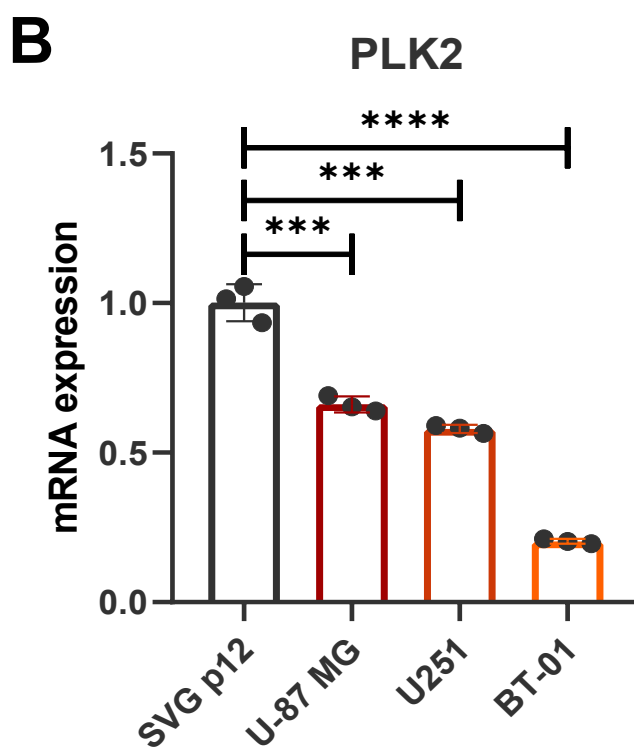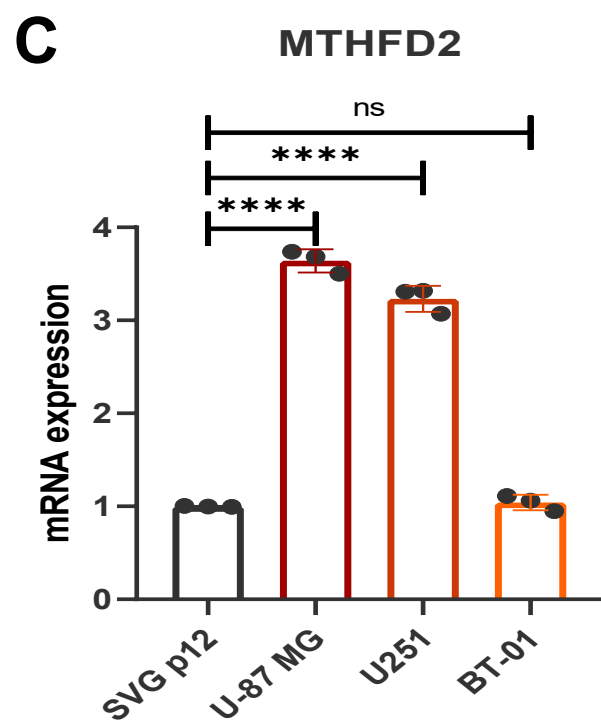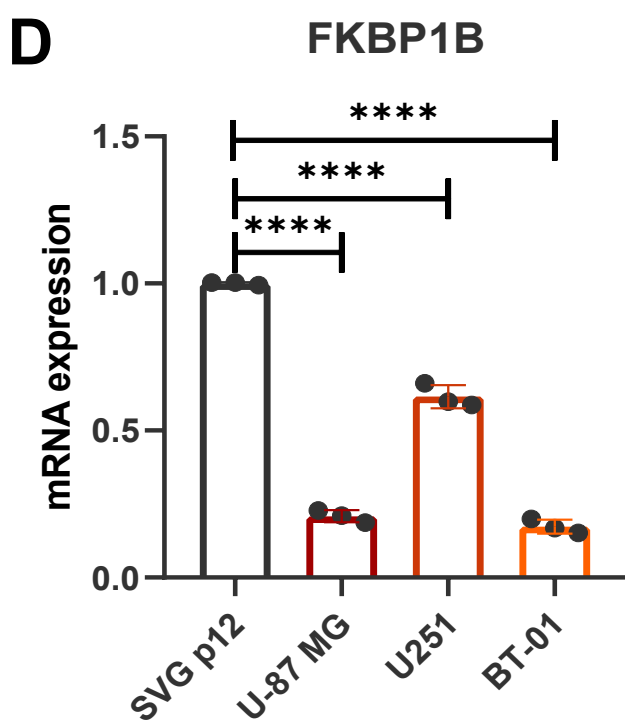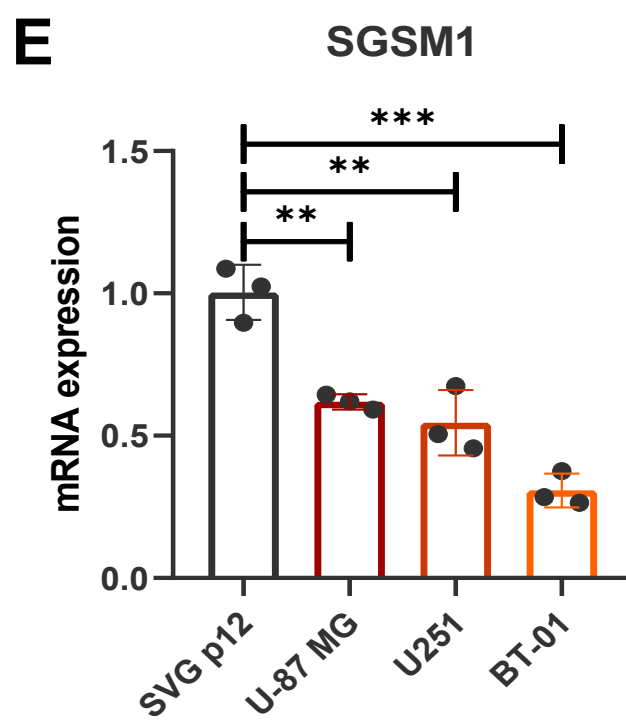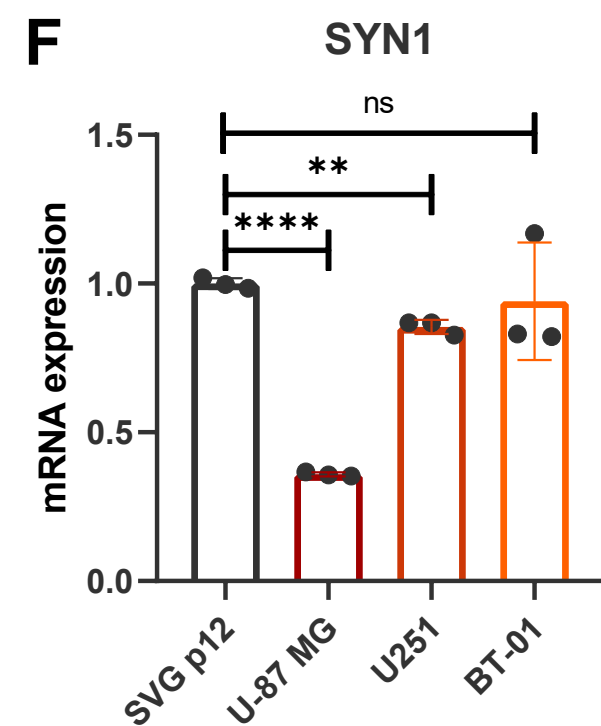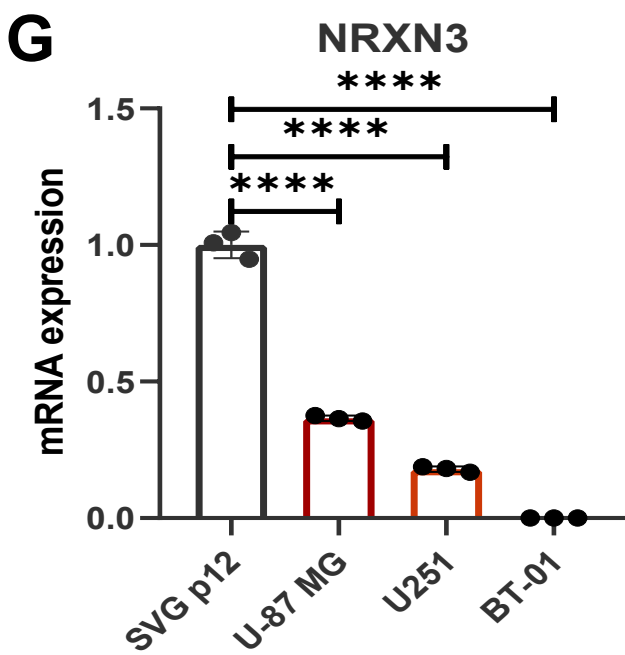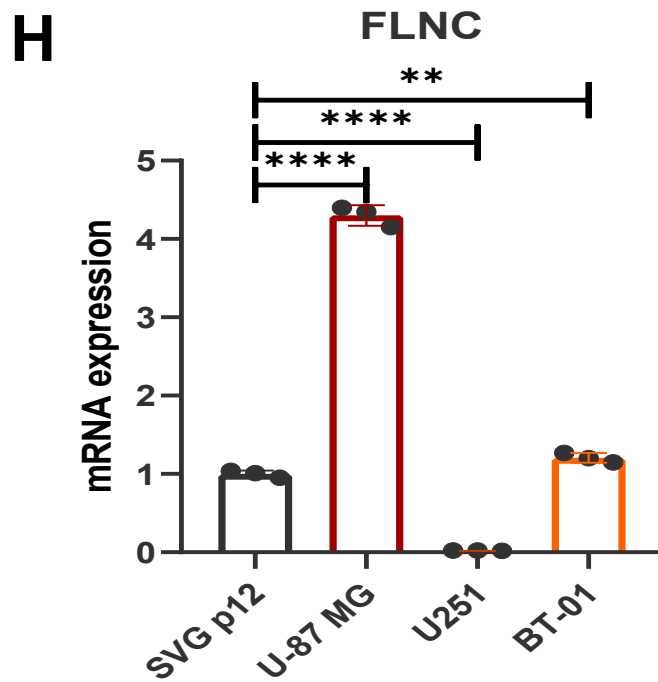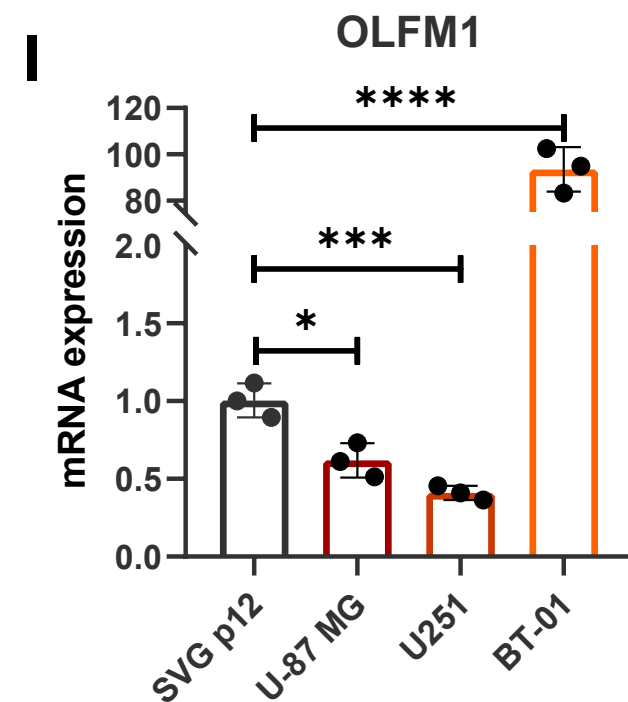

Supplement: Multimedia component 17 [file mmc17.pdf]
